# Long Term Outcomes of a Geriatric Liaison Intervention in Frail Elderly Cancer Patients

**DOI:** 10.1371/journal.pone.0143364

**Published:** 2016-02-22

**Authors:** Liesbeth Hempenius, Joris P. J. Slaets, Dieneke van Asselt, Truuske H. de Bock, Theo Wiggers, Barbara L. van Leeuwen

**Affiliations:** 1 University Center for Geriatric Medicine, University Medical Center Groningen, University of Groningen, Groningen, the Netherlands; 2 Geriatric Center, Medical Center Leeuwarden, Leeuwarden, the Netherlands; 3 Department of Geriatrics, Radboud University Medical Center, Nijmegen, the Netherlands; 4 Department of Epidemiology, University Medical Center Groningen, University of Groningen, Groningen, the Netherlands; 5 Department of Surgery, University Medical Center Groningen, University of Groningen, Groningen, the Netherlands; University of Nebraska Medical Center, UNITED STATES

## Abstract

**Background:**

The aim of this study was to evaluate the long term effects after discharge of a hospital-based geriatric liaison intervention to prevent postoperative delirium in frail elderly cancer patients treated with an elective surgical procedure for a solid tumour. In addition, the effect of a postoperative delirium on long term outcomes was examined.

**Methods:**

A three month follow-up was performed in participants of the Liaison Intervention in Frail Elderly study, a multicentre, prospective, randomized, controlled trial. Patients were randomized to standard treatment or a geriatric liaison intervention. The intervention consisted of a preoperative geriatric consultation, an individual treatment plan targeted at risk factors for delirium and daily visits by a geriatric nurse during the hospital stay. The long term outcomes included: mortality, rehospitalisation, Activities of Daily Living (ADL) functioning, return to the independent pre-operative living situation, use of supportive care, cognitive functioning and health related quality of life.

**Results:**

Data of 260 patients (intervention n = 127, Control n = 133) were analysed. There were no differences between the intervention group and usual-care group for any of the outcomes three months after discharge. The presence of postoperative delirium was associated with: an increased risk of decline in ADL functioning (OR: 2.65, 95% CI: 1.02–6.88), an increased use of supportive assistance (OR: 2.45, 95% CI: 1.02–5.87) and a decreased chance to return to the independent preoperative living situation (OR: 0.18, 95% CI: 0.07–0.49).

**Conclusions:**

A hospital-based geriatric liaison intervention for the prevention of postoperative delirium in frail elderly cancer patients undergoing elective surgery for a solid tumour did not improve outcomes 3 months after discharge from hospital. The negative effect of a postoperative delirium on late outcome was confirmed.

**Trial Registration:**

Nederlands Trial Register, Trial ID NTR 823.

## Introduction

Hospitalized elderly are at increased risk for functional decline resulting in adverse health outcomes such as mortality, prolonged hospital stay, nursing home placement and increased dependency at home. It is estimated that approximately 35% of patients aged 75 and older develop a new disability after hospitalization or suffer functional decline [1–3].

To limit functional decline after hospital stay, the prevention of delirium is of great importance. Delirium is a common and serious complication in hospitalized elderly people. It is associated with persistent functional and cognitive decline, increased morbidity and mortality, longer hospital stays, higher rates of nursing home placement and increased health-care costs [4–7]. Mortality rates vary from 4% to 20% in patients who develop delirium during their hospital stay [8,9].

We performed a randomized controlled trial to evaluate the effect of a multicomponent intervention compared to standard care, on the incidence of postoperative delirium in frail elderly cancer patients undergoing surgery for a solid tumour [10]. The intervention was targeted at risk factors for postoperative delirium: cognitive impairment, visual impairment, hearing impairment, malnutrition, pain, sleep disturbance, defecation problems, infection and impaired mobility. Delirium was chosen as the primary outcome measure because it could be determined within the intervention period during hospital stay. The intervention has not shown to be effective for preventing postoperative delirium [10]. Three months after discharge, a follow-up was performed. The follow-up measurements were focused on postoperative functional outcomes such as Activities of Daily Living (ADL) functioning, return to the independent pre-operative living situation, use of supportive care, cognitive functioning and health related quality of life, next to mortality and rehospitalisation. Most previous studies on adverse outcomes after cancer surgery in the elderly were targeted at outcomes such as postoperative complications, mortality, length of hospital stay and readmissions [11–14], while ADL functioning and quality of life (QOL) are at least as important outcomes of surgical treatment for the elderly.

In this manuscript, the long term results, three months after discharge, and the effect of postoperative delirium on long term outcomes are described. The long term outcomes included: mortality, rehospitalisation, ADL functioning, return to the independent pre-operative living situation, use of supportive care, cognitive functioning and health related quality of life.

## Methods

### Ethics statement

The study was approved by the Medical Ethical Committee of the University Medical Center Groningen, trial ID NTR 823 (**S1 and S2 Texts**). Written informed consent was obtained from the participants.

### Study design

The study, entitled Liaison Intervention in Frail Elderly (LIFE), was a multicentre, randomized clinical trial [10]. The participating centres were the University Medical Center Groningen (serving a population of three million people), the Medical Center Leeuwarden (a large teaching hospital) and Diaconessenhuis Leiden (a community hospital). All participating centres are located in the Netherlands.

The primary short term outcome of this study was the incidence of postoperative delirium up to 10 days postoperatively. The reported incidence of postoperative delirium varies widely from less than 10% to 50%. Based on these data and the fact that this study included a high‐risk population, a delirium incidence of 30% was expected in the study population. An absolute reduction of 15% was expected in the intervention group based on Inouye’s results [15]. To achieve a power of 80% with an α of 5% (one‐sided), a β of 95% and an expected drop‐out rate of 10%, it was calculated that a total of at least 294 patients would need to be included in this study.

### Participants

From June 2007 to June 2010 all consecutive patients over 65 years of age undergoing elective surgery for a solid tumour were screened with the Groningen Frailty Indicator (GFI) [16–18] at the outpatient departments of general surgery, gynaecology, ear, nose and throat medicine and maxillofacial surgery at the participating centres. The GFI is an internally consistent 15-item screening instrument used to determine an individual’s level of frailty [16,18]. The GFI is widely used in clinical practice, in outpatient settings, and in clinical studies [19–23]. It was shown that frail older persons (as identified with the GFI) had higher levels of case complexity, disability, and lower quality of life and life satisfaction [15]. Patients with a GFI score greater than 3 were regarded as frail and recruited to the LIFE study. The participants were randomly allocated to either the control group or the geriatric liaison intervention group. The randomization was stratified by tumour type. A distinction was made between tumours in the chest or abdomen and tumours elsewhere. The research nurses used an interactive voice response telephone service provided by the University Medical Center Groningen for the randomization.

Patients were excluded if the research nurse or the responsible physician estimated they were unable to complete the study protocol and follow-up schedule before inclusion (e.g. for logistical reasons or if any extra hospital visits would be too burdensome). Patients unable to fill in the questionnaires used in this study were also excluded.

### Intervention

The multicomponent intervention focused on best supportive care and the prevention of delirium. Patients in the intervention group were assessed preoperatively by a geriatric team and monitored during their hospital stay. As the three participating centres are heterogeneous and this could cause variance in how the intervention was conducted, checklists were used to standardize the intervention as much as possible.

The geriatric team was supervised by a geriatrician, and helped devise the individual care plan. The preoperative comprehensive geriatric assessment by a geriatrician consisted of a medical history, physical examination and follow-up examinations on indication resulting in an individual treatment plan, with specific attention to patient-related risk factors for delirium.

During their hospital stay, the patients in the intervention group were assessed daily by a geriatric nurse. If a problem was encountered, the geriatric nurse or geriatrician contacted the treatment team to discuss the proposed intervention and establish a treatment plan, checking daily to determine whether the advice had been followed. For a detailed description of the intervention we refer to [10].

#### Standard care

Patients in the usual-care group received standard care, meaning that additional geriatric care was only provided at the request of the treating physician.

#### Surgical procedure

Surgical procedures were divided into three categories: minor, intermediate and major according to the duration of the operation and the localization of the tumour (intracavitary versus superficial (Table 1).

**Table 1:** Classification of the type of surgery by duration of the procedure and tumour localization.

Surgery load	Tumour localization
Minor	Breast and skin
Intermediate	Vulva, cervix, endometrium, uterus, head/neck and retroperitoneum
Major	Gastrointestinal, liver, pancreas, lung, ovary, oropharynx, larynx and intra-abdominal sarcoma

### Long term outcomes

The long term outcomes considered in the here presented analyses were mortality, rehospitalisation, ADL functioning, return to the independent pre-operative living situation, supportive care, cognitive functioning, and health related quality of life. The measurement instruments that were used for these outcomes are described in the assessments section.

#### Assessments

The baseline assessment was completed by the research nurses at least 24 hours before surgery and was performed prior to randomization. Data on long-term outcomes were collected by the research nurses 3 months following hospital discharge during a telephone interview or a home visit, between August 2007 and November 2010.

At baseline, demographic data were collected. Both the baseline assessment and the follow-up assessment included the measurement of the health related quality of life by the Physical Component Summary measure (PCS) and the Mental Component Summary measure (MCS) of the Short Form-36 (SF-36) score [24–26]; basic ADL functioning by the Care Dependency Scale (CDS) [27] and cognitive functioning by the Mini-Mental State Examination (MMSE) [28]. Data regarding the living situation and supportive care (domestic help, care assistance and informal care) were also collected.

To screen for delirium during hospital stay, the Delirium Observation Scale (DOS) was used in both groups [29]. The DOS was recorded three times a day, up to 10 days postoperatively. In the case of a mean DOS score ≥ 3 (possible delirium) a geriatrician or psychiatrist examined the patient to confirm the diagnosis according to the criteria of the *Diagnostic and Statistical Manual of Mental Disorders*, *Fourth Edition* (DSM IV).

A paper-based standardized form was used to collect data. Data were entered into Oracle Clinical Remote Data Capture program by trained research nurses. After entry, the data were checked by an independent individual. The research nurses were not blinded to the group the patients had been assigned to.

#### Definition of long term outcomes

For the current analysis all long term outcomes were considered as binaries. ADL functioning, was categorized in a lower score at 3-month follow-up compared to the baseline the baseline score (“decreased”) versus a same or higher score (“same/ increased”). Use of supportive care was dichotomized in an increased number of hours supportive care per week at 3-month follow-up compared to baseline (“increased”) versus the same or a decreased number of hours supportive care (“same/ decreased”). Cognitive functioning was categorized as MMSE score decreased ≥ 2 points at 3-month follow-up versus baseline versus MMSE score same or increased. Health related quality of life was dichotomized as a decreased score on the SF-36 physical and mental component scale at 3 month follow up versus baseline (“decreased) versus a same or increased score (“same/ increased”).

### Statistical analysis

Differences in baseline characteristics between the groups were examined using a Fisher exact test for nominal variables and a two-sample Smirnov test for ordinal or continuous variables.

To examine the effectiveness of the intervention as compared to standard care on the long term outcomes at 3 months follow up, univariate binary logistic regression analysis was used and Odds Ratios (ORs) with a 95% Confidence Intervals (CIs) were estimated, where the intervention was considered as independent and the long-term outcomes were considered as dependents. There was no pre-determined hierarchy between the long term outcomes. The primary outcome of this study, postoperative delirium, was evaluated previously [10]. In case of a p-value < 0.05, correction for multiple testing was performed.

The effect of postoperative delirium (independent variable) on the outcomes at 3 months follow up (dependent variable) was also calculated using univariate binary logistic regression analysis.

IBM SPSS Statistics Version 20 was used for the statistical analysis.

## Results

**Fig 1:**
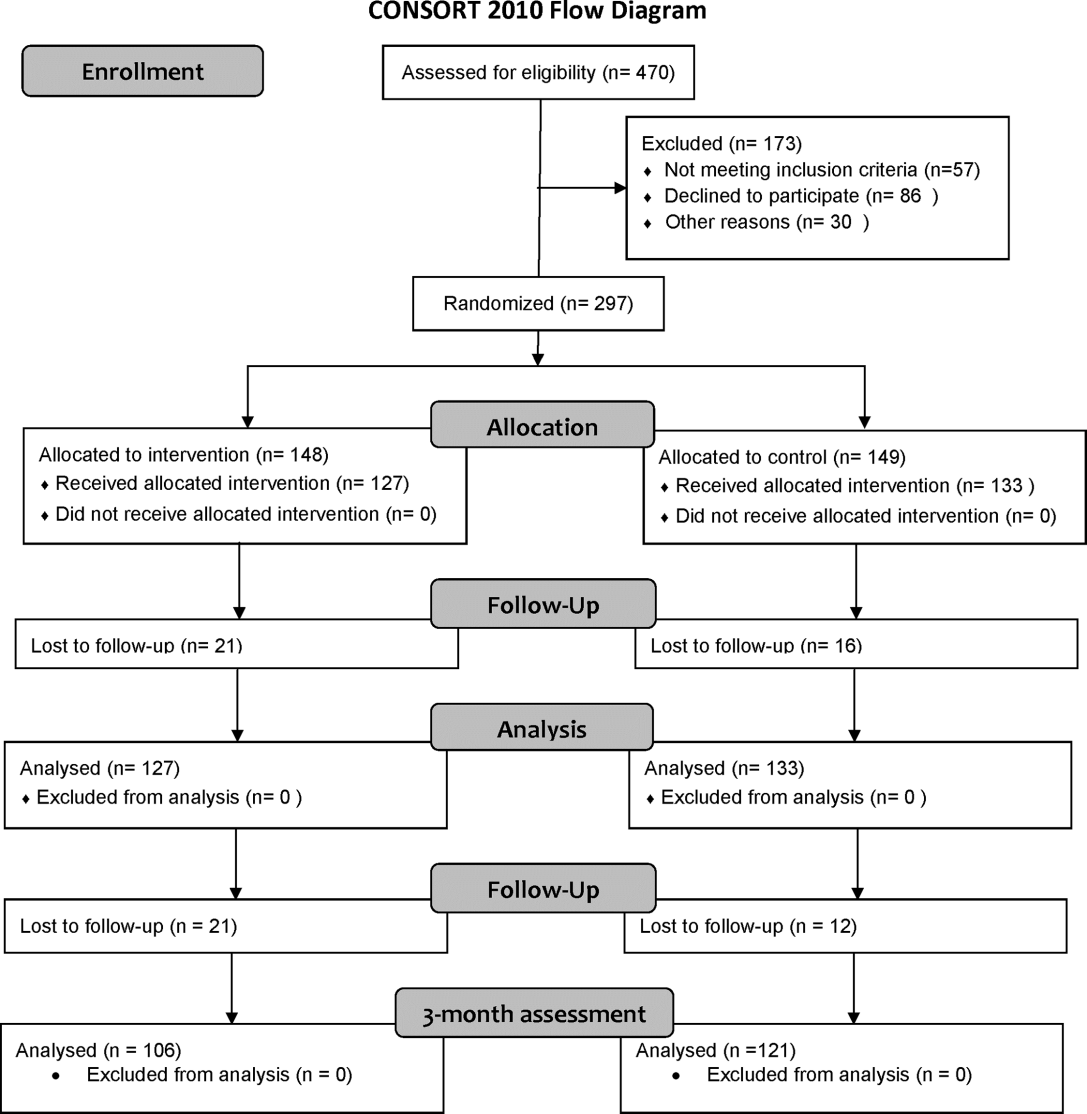
CONSORT diagram for the study.

A description of the flow of participants through each stage of the LIFE study was presented in a previous article [10]. Of the 260 patients who were followed during hospital stay, 33 were lost to follow-up at the time of the 3-month assessment: 14 died during hospital stay, 12 died before follow-up assessment, seven withdrew informed consent post discharge. Therefore the final sample size for this study was 227: 106 intervention group and 121 usual-care group (Fig 1, lower part). There were no significant differences between the groups at discharge (Table 2).

**Table 2:** Characteristics of the patients at discharge according to study group.

Characteristic	Intervention group (n = 127)	Usual-care group (n = 133)	P-value
**Age (years), mean (SD)**	77.37 (6.88)	77.42 (7.71)	0.49†
**Female, n (%)**	76 (59.8)	85 (63.9)	0.53‡
**Type of surgery**∫**, n (%)**			0.54‡
Minor	32 (25.2)	33 (24.8)	
Intermediate	20 (15.7)	28 (21.1)	
Major	75 (59.1)	72 (54.1)	
Comorbidities*, n (%)			0.47‡
≤ 2	51 (40.2)	55 (41.4)	
>2	76 (59.8)	78 (58.6)	
**Living situation, n (%)**			0.10‡
independent	113 (89.0)	110 (82.7)	
*alone*	55 (43.3)	53 (39.8)	
*with others*	58 (45.7)	57 (42.9)	
dependent	14 (11.0)	23 (17.3)	
*protected housing*	0 (0.0)	4 (3.0)	
*home for the elderly*	11 (8.7)	16 (12.0)	
*nursing home*	3 (2.4)	3 (2.3)	
**Supportive care, n (%)**			
Domestic help			0.46‡
*No*	60 (47.2)	61 (46.2)	
*Yes*	67 (52.8)	71 (53.8)	
*Missing*	0	1	
Care assistance			0.40‡
*No*	87 (69.0)	94 (71.2)	
*Yes*	39 (31.0)	38 (28.8)	
*Missing*	1	1	
Informal care			0.49‡
*No*	68 (54.0)	70 (53.0)	
*Yes*	58 (46)	62 (47.0)	
Missing	1	1	
**Care Dependency Score, mean (SD)**	72.49 (8.52)	74.23 (6.97)	0.27†
**Mini Mental State Examination, mean (SD)**	26.97 (2.47)	26.51 (3.74)	0.97†
Missing	19	31	
**Short Form-36, mean (SD)**			
Physical component summary measure	48.36 (9.07)	49.32 (7.02)	0.17†
Mental component summary measure	44.69 (8.79)	44.38 (8.42)	0.98†

†Kolmogorov-Smirnov test.

‡ Fisher’s exact test.

∫Surgery load: Major = gastrointestinal, liver, pancreas, lung, ovary, oropharynx, larynx and intra-abdominal sarcoma. Intermediate = vulva, cervix, endometrium, uterus, head/neck and retroperitoneum. Minor = breast and skin.

*Comorbidities = diabetes, COPD, hypertension, myocardial infarction, other cardiovascular disorders, neurological disorders, cerebrovascular disorders, hearing and vision problems, memory problems in daily life, psychiatric disorders or musculoskeletal disorders.

### Long term outcomes

The results of the logistic regression analyses for the outcome variables are shown in Table 3. There were no significant differences between the intervention and usual-care group for any of the outcomes.

**Table 3:** Univariate Logistic regression analyses for the effectiveness of the intervention compared to standard care on the long term outcomes (intervention group versus control group).

	Mortality Yes	Mortality No	Missing cases	OR (95% CI)
Control group	9 (6.8)	124 (93.2)		1
*During hospital stay*	4			
*After discharge*	5			
Intervention group	17 (13.4)	110 (86.6)		2.13 (0.91–4.97)
*During hospital stay*	10			
*After discharge*	7			
	**Hospital readmission Yes**	**Hospital readmission No**		
Control group	22 (18.3)	98 (81.7)	1	1
Intervention group	24 (22.9)	81 (77.1)	1	1.32 (0.69–2.53)
	**ADL functioning Decreased**	**ADL functioning Same/ increased**		
Control group	68 (56.2)	53 (43.8)		1
Intervention group	64 (60.4)	42 (39.6)		1.19 (0.70–2.02)
	**No return to independent preoperative living situation**	**Return to independent preoperative living situation**		
Control group	9 (8.9)	92 (91.1)		1
Intervention group	15 (16.5)	76 (83.5)		2.02 (0.84–4.87)
	**Use of supportive care Increased**	**Use of supportive care Same/ decreased**		
***Domestic help***				
Control group	38 (32.2)	80 (67.8)	4	1
Intervention group	33 (32.4)	69 (67.6)	4	1.01 (0.57–1.78)
***Care assistance***				
Control group	39 (33.3)	78 (66.7)	4	1
Intervention group	42 (41.2)	60 (58.8)	4	1.40 (0.81–2.43)
***Informal care***				
Control group	37 (31.6)	80 (68.4)	4	1
Intervention group	39 (38.2)	63 (61.8)	4	1.34 (0.57–1.78)
	**Cognitive functioning decreased**	**Cognitive functioning same/ increased**		
Control group	9 (14.1)	55 (85.9)	57	1
Intervention group	15 (23.1)	50 (76.9)	41	1.83 (0.74–4.56)
	**Health related quality of life decreased** *SF-36 Physical component summary measure*	**Health related quality of life same/ increased** *SF-36 Physical component summary measure*		
Control group	80 (66.7)	40 (33.3)	1	1
Intervention group	63 (60)	42 (40)	1	1.33 (0.77–2.30)
	**Health related quality of life decreased** *SF-36 Mental component summary measure*	**Health related quality of life same/ increased** *SF-36 Mental component summary measure*		
Control group	53 (44.2)	67 (55.8)	1	1
Intervention group	51 (48.6)	54 (51.4)	1	0.84 (0.50–1.42)

### Influence of postoperative delirium on long term outcomes

In total, 227 patients were analysed for the long term outcomes of delirium. A postoperative delirium occurred in 26 of these patients (11.5%). Delirium increased the risk of a decline in ADL functioning (OR: 2.65, 95% CI: 1.02–6.88) resulting in an increased need for care assistance (OR: 2.45, 95% CI: 1.02–5.87) and a decreased chance to return to the independent preoperative living situation (OR: 0.18 (0.07–0.49). These results are presented in Table 4.

**Table 4:** Univariate logistic regression analyses for the influence of postoperative delirium on 3-month outcomes.

	n (%)	n (%)	Missing cases	OR (95% CI)
	Mortality Yes	Mortality No		
No postoperative delirium	21 (9.5)	201 (90.5)		1
*During hospital stay*	10			
*After discharge*	11			
Postoperative delirium	5 (16.1)	26 (83.9)		1.90 (0.66–5.48)
*During hospital stay*	4			
*After discharge*	1			
	**Hospital readmission Yes**	**Hospital readmission No**		
No postoperative delirium	43 (21.5)	157 (78.5)	1	1
Postoperative delirium	3 (12)	22 (88)	1	0.50 (0.14–1.74)
	**ADL functioning Decreased**	**ADL functioning Same/ increased**		
No postoperative delirium	112 (55.7)	89 (44.3)		1
Postoperative delirium	20 (76.9)	6 (23.1)		2.65 (1.02–6.88)*
	**No return to independent preoperative living situation**	**Return to independent preoperative living situation**		
No postoperative delirium	17 (9.9)	155 (90.1)		1
Postoperative delirium	8 (38.1)	13 (61.9)		0.18 (0.07–0.49)*
	**Use of supportive care Increased**	**Use of supportive care Same/ decreased**		
***Domestic help***				
No postoperative delirium	65 (33.0)	132 (67)	4	1
Postoperative delirium	6 (26.1)	17 (73.9)	3	0.72 (0.27–1.90)
***Care assistance***				
No postoperative delirium	68 (34.7)	128 (65.3)	5	1
Postoperative delirium	13 (56.5)	10 (43.5)	3	2.45 (1.02–5.87)*
***Informal care***				
No postoperative delirium	68 (34.7)	128 (65.3)	5	1
Postoperative delirium	8 (34.8)	15 (65.2)	3	1.00 (0.41–2.49)
	**Cognitive functioning decreased**	**Cognitive functioning same/ increased**		
No postoperative delirium	21 (18.1)	95 (81.9)	85	1
Postoperative delirium	3 (23.1)	10 (76.9)	3	1.36 (0.34–5.36)
	**Health related quality of life decreased *SF-36 Physical component summary measure***	**Health related quality of life same/ increased *SF-36 Physical component summary measure***		
No postoperative delirium	123 (61.5)	77 (38.5)	1	1
Postoperative delirium	20 (80)	5 (20)	1	2.26 (0.96–5.36)
	**Health related quality of life decreased *SF-36 Mental component summary measure***	**Health related quality of life same/ increased *SF-36 Mental component summary measure***		
No postoperative delirium	88 (44)	112 (56)	1	1
Postoperative delirium	16 (64)	9 (36)	1	(0.90–6.95)

*significant difference

## Discussion

Three months after discharge from hospital no benefit could be detected from a geriatric liaison intervention targeted at risk factors for postoperative delirium in frail elderly patients undergoing surgery for a solid tumour. Because postoperative delirium is a known risk factor for functional decline after hospital stay [4–7], we, a priori, hypothesized that prevention of postoperative delirium would result in decreased risk for adverse outcomes after hospitalisation. Other studies have shown varying results of multicomponent delirium prevention interventions on long term outcomes [15, 30, 31].

The low delirium incidence rates found in the LIFE study (14.3% in the control group versus 9.4% in the intervention group) may have been of crucial importance for our negative results [10]. This resulted in an underpowered study. The intervention appeared not to be effective in preventing delirium in the population under study and showed consequent no effect on long term results.

Factors that probably contributed to the low delirium incidence rate in our study are the exclusion of patients with severe cognitive impairment (high risk for postoperative delirium) and the inclusion of patients undergoing superficial surgery (low risk for postoperarive delirium). Furthermore the low delirium incidence rate implies a high standard of care for frail elderly patients in the participating hospitals before the start of the study and the introduction of the Delirium Observation Scale (DOS) [29] on the wards to screen for delirium may have ensured increased alertness among medical staff for the prevention of postoperative delirium, in both the intervention and control group.

The long term results of this type of studies may be influenced by a wash-out effect due to interventions performed after discharge and outside the study protocol. Probably, continuation of in hospital interventions after discharge might overcome this, although, little is known about the effect of prolonged interventions in elderly patients who were hospitalized. One study showed a significantly decreased mortality in older cancer patients after a 4 weeks lasting intervention post discharge [32].

Up to 50% of elderly patients suffer functional decline after hospitalization resulting in a decline in health-related quality of life and loss of independence in (I)ADL functioning [1,33]. In our study, also a considerable part of patients suffered a postoperative decline in ADL functioning (60.4% in the intervention group versus 56.2% in the control group) and health related QOL (physical component: 60% in the intervention group versus 66.7% in the control group; mental component: 48.6% in the intervention group versus 44.2% in the control group) (See Table 2).

For the frail elderly surgical oncology patients participating in the LIFE study, postoperative delirium was a risk factor for functional decline after discharge. Delirium was associated with: an increased risk of a postoperative decline in ADL functioning, an increased use of care assistance and a decreased chance to return to the independent preoperative living situation. Only 26 patients that had developed postoperative delirium versus 201 nondelirious patients were tested in this analysis. Our data confirm that a postoperative delirium is a sign of increased (brain) vulnerability associated with poorer prognosis [34]. Therefore, targeting preventive interventions at those elderly at risk for (postoperative) delirium remains a major concern in minimizing functional decline after hospitalization.

The results shown in this manuscript concern a post hoc analysis. The effectiveness of a geriatric liaison intervention as well as the effect of a postoperative delirium on the outcomes 3 months postoperative (dependent variables) were explored using binary logistic regression analysis. In view of the nature of the analysis (post hoc) and the number of dependent variables tested (11 in total), the result should be interpreted with some caution.

In conclusion, the lower than expected delirium incidence rate and the high standard of basic care may have influenced the long term results. The association between postoperative delirium and functional decline after hospitalization was confirmed in the population under study. Therefore prevention of postoperative delirium seems one of the ways to limit functional decline after surgery in this patient group.

## Supporting Information

S1 Text Study Protocol. (DOC)

S2 Text CONSORT checklist. (DOC)
