# Supplementary material for: Long Term Outcomes of a Geriatric Liaison Intervention in Frail Elderly Cancer Patients
Source: PLoS One. 2016 Feb 22;11(2):e0143364. doi: 10.1371/journal.pone.0143364 (PMC4762573; doi:10.1371/journal.pone.0143364)
Supplement: S1 Text — (DOC) [file pone.0143364.s001.doc]

**A randomized controlled trial of geriatric liaison intervention in frail surgical oncology patients**

**A randomized controlled trial of geriatric liaison intervention in frail surgical oncology patients**

| **Protocol ID** | **Geriatric intervention** |
| --- | --- |
| **Short title** |  |
| **Version** |  |
| **Date** | **October 2006** |
| **Coordinating investigator/project leader** | ***Barbara van Leeuwen***  [***blvleeuwen@hetnet.nl***](mailto:blvleeuwen@hetnet.nl)  ***050-5891968*** |
| **Principal investigator(s) (in Dutch: hoofdonderzoeker/uitvoerder)**  ***Multicenter research: per site*** | ***Prof T. Wiggers. Afdeling Heelkunde, Universitair Medisch Centrum Groningen, Postbus 4195, 9700 ED Groningen***  ***Prof J Slaets, Geriatrie Universitair Medisch Centrum Groningen***  ***Prof HJ Hoekstra, Chirurgie, Universitair Medisch Centrum Groningen***  ***Dr T de Bock, Epidemiologie , Universitair Medisch Centrum Groningen***  ***Dr. R. Vree, Afdeling Heelkunde, Diaconessenhuis, Houtlaan 55, 2334 CK Leiden***  ***Dr. J.P.E.N. Pierie, Afdeling Heelkunde, Medisch Centrum Leeuwarden, Henri Dunantweg 2, postbus 888, 8901BR Leeuwarden*** |
|  |  |
| **Sponsor (in Dutch: verrichter/opdrachtgever)** | ***ZonMw, Nederlandse organisatie voor gezondheidsonderzoek en zorginnovatie***  ***Postbus 93245, 2509 AE Den Haag, tel: 070-3495111, fax: 070-3495100, info@zonmw.nl >*** |
|  |  |
| **Independent physician(s)** | ***Dr.JT.M. Plukker , Afdeling Heelkunde, Universitair***  ***Medisch Centrum Groningen*** |
|  |  |
|  |  |
|  |  |
|  |  |
|  |  |
|  |  |
|  |  |

**PROTOCOL SIGNATURE SHEET**

| **Name** | **Signature** | **Date** |
| --- | --- | --- |
| **For non-commercial research,**  **Head of Department:**  **Prof. Dr. T. Wiggers** |  |  |
| **Coordinating Investigator/Project leader/Principal Investigator:**  **Dr. B.L. van Leeuwen** |  |  |
| **Dr. J.P.E.N. Pierie** |  |  |
| **Dr. R. Vree** |  |  |

**TABLE OF CONTENTS**

1. SUMMARY 8

2. INTRODUCTION AND RATIONALE 9

3. OBJECTIVES [11](#__RefHeading___Toc149202999)

4. STUDY DESIGN [12](#__RefHeading___Toc149203000)

i. Inclusion phase 1: 3 months [12](#__RefHeading___Toc149203001)

ii. Inclusion phase 2: 3-33 months [12](#__RefHeading___Toc149203002)

iii. Follow up and analysis phase 3: 33-36 months [12](#__RefHeading___Toc149203003)

5. STUDY POPULATION [13](#__RefHeading___Toc149203004)

5.1 Population (base) [13](#__RefHeading___Toc149203005)

5.2 Inclusion criteria [13](#__RefHeading___Toc149203006)

5.3 Exclusion criteria [13](#__RefHeading___Toc149203007)

5.4 Sample size calculation [13](#__RefHeading___Toc149203008)

6. TREATMENT OF SUBJECTS [14](#__RefHeading___Toc149203009)

6.1 Investigational treatment [14](#__RefHeading___Toc149203010)

7. METHODS [18](#__RefHeading___Toc149203011)

7.1 Study parameters/endpoints [18](#__RefHeading___Toc149203012)

7.1.1 Main study parameter/endpoint [18](#__RefHeading___Toc149203013)

7.1.2 Secondary study parameters/endpoints [18](#__RefHeading___Toc149203014)

7.2 Randomisation, blinding and treatment allocation [18](#__RefHeading___Toc149203015)

7.3 Study procedures [19](#__RefHeading___Toc149203016)

During treatment [19](#__RefHeading___Toc149203017)

After the end of treatment (Follow-up): [20](#__RefHeading___Toc149203018)

3 months postoperatively the following data will be recorded [20](#__RefHeading___Toc149203019)

7.4 Withdrawal of individual subjects 22

7.5 Replacement of individual subjects after withdrawal 22

7.6 Follow-up of subjects withdrawn from treatment 22

8. SAFETY REPORTING 23

8.1 Section 10 WMO event 23

8.2 Adverse and serious adverse events 23

8.3 Follow-up of adverse events 24

8.4 Data Safety Monitoring Board (DSMB) 24

9. STATISTICAL ANALYSIS 25

9.1 Descriptive statistics 25

10. ETHICAL CONSIDERATIONS 26

10.1 Regulation statement 26

10.2 Recruitment and consent 26

10.3 Benefits and risks assessment, group relatedness 26

10.4 Compensation for injury 27

11. ADMINISTRATIVE ASPECTS AND PUBLICATION 27

11.1 Handling and storage of data and documents 27

11.2 Amendments 27

11.3 Annual progress report 27

11.4 End of study report 27

11.5 Public disclosure and publication policy 28

12. REFERENCES 29

**LIST OF ABBREVIATIONS AND RELEVANT DEFINITIONS**

| **ABR** | **ABR form (General Assessment and Registration form) is the application form that is required for submission to the accredited Ethics Committee (ABR = Algemene Beoordeling en Registratie)** |
| --- | --- |
| **AE** | **Adverse Event** |
| **AR** | **Adverse Reaction** |
| **CA** | **Competent Authority** |
| **CCMO** | **Central Committee on Research Involving Human Subjects** |
| **CV** | **Curriculum Vitae** |
| **DSMB** | **Data Safety Monitoring Board** |
| **EU** | **European Union** |
| **EudraCT** | **European drug regulatory affairs Clinical Trials GCP Good Clinical Practice** |
| **IB** | **Investigator’s Brochure** |
| **IC** | **Informed Consent** |
| **IMP** | **Investigational Medicinal Product** |
| **IMPD** | **Investigational Medicinal Product Dossier** |
| **METC** | **Medical research ethics committee (MREC); in Dutch: medisch ethische toetsing commissie (METC)** |
| **(S)AE** | **Serious Adverse Event** |
| **SPC** | **Summary of Product Characteristics (in Dutch: officiële productinfomatie IB1-tekst)** |
| **Sponsor** | **The sponsor is the party that commissions the organisation or performance of the research, for example a pharmaceutical**  **company, academic hospital, scientific organisation or investigator. A party that provides funding for a study but does not commission it is not regarded as the sponsor, but referred to as a subsidising party.** |
| **SUSAR** | **Suspected Unexpected Serious Adverse Reaction** |
| **Wbp** | **Personal Data Protection Act (in Dutch: Wet Bescherming Persoonsgevens)** |
| **WMO** | **Medical Research Involving Human Subjects Act (Wet Medisch-wetenschappelijk Onderzoek met Mensen** |

1. **SUMMARY**

**Rationale:** It has been shown that a multicomponent intervention can decrease the occurrence of delirium in older patients (Inouye et al 1999). Geriatric liaison teams are well trained in implementing best-supportive care programs for elderly. In standard care geriatricians are not involved in pre-operative screening of patients and perioperative care and will only be consulted after major complications (delirium) or functional losses have occurred.

**Objective**: The primary objective of this study is to show that early detection of geriatric patients at risk of preventable functional decline following a surgical procedure under general anesthesia for a solid tumor, combined with a geriatric liaison intervention will decrease the occurrence of delirium and consequent morbidity and mortality, without an increase in costs.

**Study design:** This is a multicenter prospective randomized clinical trial.

**Study population:** Patients over 65 years of age admitted to the Department of Surgery of the participating centres for the surgical or combined cancer treatment (surgery / radiation /chemotherapy / hormonal therapy) of a solid tumor will be included in this study

**Intervention**: The intervention entails participation of a geriatric nurse and geriatrician in the perioperative treatment of the oncogeriatric surgical patient.

**Main study parameters/endpoints:** The main endpoint is the cumulative incidence of delirium (measured with the Delirium Observation Scale and the DSM IV criteria) up to 10 days postoperatively. Secondary endpoints are: returning to the pre-operative living situation within 3 months postoperatively,the Physical Component Summary measure (PCS) of the SF-36, the Mental Component Summary measure (MCS) of the SF-36, complications during hospital stay including mortality, care Dependence Scale at discharge. Direct health care and non-health care costs will be used as economic indicators

**Nature and extent of the burden and risks associated with participation, benefit and group relatedness:** The participating patients will have to complete 2 questionnaires at inclusion in the study which will take about 30 minutes in total. During their hospital stay they will be asked to complete several questionnaires which will take 15 minutes daily on average. Also 3 months postoperatively they will be asked to complete a questionnaire which takes 15-30 minutes on average. The Hb value will be taken from the routine blood samples and no extra blood samples or diagnostic tests will be performed on the participating patients unless their medical condition requires this (e.g. to rule out dehydration). Although changes in diet or medication may be made in the study group these are not expected to cause an extra burden or discomfort to the participating patients. No experimental drugs will be used during this study.

2. INTRODUCTION AND RATIONALE

Cancer is a disease of the elderly: 50% of all new cases of cancer and 60% of all cancer deaths occur in patients over 65 years. It is expected that the prevalence of cancer in the Dutch population will increase with 35% (breast cancer) to 60% (lung cancer) in the next 20 years with an increase in the absolute number of patients older than 65 years. The estimate number of cancer patients older than 65 in The Netherlands is 91.000. For 2020 the prediction will be 137.000 patients. About 57% of the patients with a solid tumor are treated with surgery, mostly as part of a combined modality treatment including (neo) adjuvant radiation and/or adjuvant chemotherapy and/or hormonal treatment. Although postoperative mortality is acceptable in selected elderly patients (Damhuis 2005), fewer chances of proper diagnosis and good quality of care are currently offered to the older cancer patients (Repetto, 2003; Vercelli et al 2000; Audisio et al 2004; Fentiman et al, 1996). Hospitalization of older patients is often associated with the development of disability that is potentially preventable. It is estimated that approximately 35% of patients aged 75 and older develop a new disability after hospitalization or suffer functional declines. This holds true for the Netherlands (Olde Rikkert, 2004), as well as for other countries (e.g., Rothschild et al., 2000; Sager et al., 1996). Institutionalization of these patients leads to further costs, not to mention loss of quality of life. Moreover, it is estimated that a considerable part of these adverse effects in hospitalized patients are preventable. Estimates range from 35% (e.g., Olde Rikkert, 2004) to 65% (e.g., Brennan et al., 1991). It is paramount to early identify the most complex cases at risk of developing new functional losses in an attempt to prevent their occurrence during the cancer treatment and the course of hospitalization. (Wymenga et al, 2001). After an uneventful recovery the prognosis of cancer patients is similar to the recovery in young patients. More patients will be able to receive proper adjuvant treatment. As 50% to 70% of elderly patients in hospital do not have an increased risk for adverse outcomes due to geriatric problems, geriatric interventions should be targeted not to elderly per se but to frail elderly only. At the University Medical Center Groningen a case-finding instrument based on these principles has been developed. The Groningen Frailty Indicator (GFI) (Schuurmans et al., 2004; Steverink et al., 2001; Slaets, 2004) has been applied in a wide range of patients and elderly populations. We did a prospective study on 85 consecutive admissions for oncological surgery in the UMCG. 30% of these patients had a GFI greater than 3 (mean score 3, median score 2). About one third of the patients over 65 years old in this study will have a GFI score greater than 3. They are considered as frail elderly. The effects of intervention will be studied only in this subgroup of patients in comparison to standard care.

The importance of delirium.

Delirium is a common psychiatric disorder in hospitalized elderly. It is a serious and potentially preventable medical condition. It is an acute confusional state characterized by an alteration of consciousness with reduced ability to focus, sustain or shift attention (Francis 1990). Reported incidence in literature varies widely from less than 10 up to 50% of hospitalized patients and it is often misdiagnosed. The occurrence of postoperative delirium in elderly patients is associated with an increase in morbidity and mortality (Van Hemert 1994). Delirium is also a costly disorder, increasing length of hospital stay and healthcare costs by up to 25% (Franco 2001). Based on literature it seems fair to assume a 30% incidence of postoperative delirium in frail elderly cancer patient postoperatively. There are two steps in the process of diagnosing delirium. The first step is to screen every patient at risk by nurses on a day tot day basis. Nurses play a crucial role in the recognition of delirium as they have frequent contacts with the patients and are in a good position to observe behavioural changes. We will use the Delirium Observation Scale (DOS) as a screening instrument for delirium. This observation rated screening scale was designed for the early recognition of delirium based on observations by the nurses during regular care. The DOS has a high internal consistency with a Cronbach’s alpha 0,92 to 0,96 (M. Schuurmans et.al). The sensitivity of the algorithm based on 13 DOS-items against the clinical DSM-IV diagnosis was 100% with a specitivity of 68%. (Schuurmans). The negative predictive value of the DOS algorithm in this study was also 100%. The nurses in the study will be trained to use the DOS. A score higher than 3 indicates delirium. The second step is to make the formal diagnosis of delirium by a medical specialist (geriatrician or psychiatrist) on the base of the DSM-IV criteria. The severity of delirium will be rated by the Delirium Rating Scale (DRS-R-98)

Motivation for the geriatric intervention

It has been shown that a multicomponent intervention can decrease the occurrence of delirium in older patients (Inouye et al 1999). Geriatric liaison teams are well trained in implementing best-supportive care programs for elderly. In standard care geriatricians are not involved in pre-operative screening of patients and perioperative care and will only be consulted after major complications (delirium) or functional losses have occurred. Combining the disease-oriented approach of surgeons with the patient-oriented approach of geriatricians we expect to decrease the occurrence of postoperative delirium in the frail elderly cancer patient treated for a solid tumor from 30% to 15%.

# 3. OBJECTIVES

The primary objective of this study is to show that early detection of geriatric patients at risk of preventable functional decline following a surgical procedure under general anesthesia for a solid tumor, combined with a geriatric liaison intervention will decrease the occurrence of delirium and consequent morbidity and mortality, without an increase in costs.

# 4. STUDY DESIGN

This is a multicenter prospective randomized clinical trial.

Patients eligible for the trial will be randomized to receive either perioperative intervention by the geriatric team or standard care.

**Stratification**

Randomization will be stratified by:

- tumor type e.g. a distinction will be made between tumors located in the chest (lung) or abdomen (liver, gastro-intestinal and pancreas) vs other tumors (breast, sarcoma, melanoma, thyroid).

**Trial phases**

### Inclusion phase 1: 3 months

During the first 3 months all patients above 65 years will be assessed with the Groningen Frailty Index. For the patients with an index greater than 3 the geriatric liaison teams will be organized and the necessary steps to make the work of the teams comparable will take place. The geriatric liaison will prevent or detect adverse events in an earlier phase than usual.

### Inclusion phase 2: 3-33 months

In this period the randomization of the patients with a high level of frailty will take place. There will be no inclusions in this period for patients with a low level of frailty. We will randomize 132 patients in the care as standard group and 132 patients in the intervention group.

### Follow up and analysis phase 3: 33-36 months

Since the primary outcome is delirium, the analysis can start immediately after inclusion of the last patient. However, during the last period the follow up assessments will be completed in order to score the secondary endpoints. The final analysis and scientific output of the primary and secondary outcomes of the study will be completed.

# 5. STUDY POPULATION

## 5.1 Population (base)

Patients over 65 years of age admitted to the Department of Surgery of the participating centres for the surgical or combined cancer treatment (surgery / radiation /chemotherapy / hormonal therapy) of a solid tumor will be included in this study.

## 5.2 Inclusion criteria

- a score greater than 3 on the GFI.
- surgery is scheduled more than 24 hours after inclusion in the study as we feel this is the time necessary for the geriatric team to plan their perioperative measures
- surgery under general anesthesia.
- written informed consent given according to local regulations.

Patients can only be randomized in this trial once.

## 5.3 Exclusion criteria

- any psychological, familial, sociological or geographical condition potentially hampering compliance with the study protocol and follow-up schedule.
- Patient unable to comply with the outcome questionnaires

## 5.4Sample size calculation

Sample size and stopping rules were computed using EAST- software.

The incidence of delirium in our population is assumed 30%. We expect to find an absolute reduction of 15 %. Group sample sizes of 2x132 to achieve 80% power to detect a difference of 0.15 with a significance level of 0.05 (one sided). With a drop out rate of 10% 294 patients are needed. There are at least 120 patients aged 65 and over treated for solid tumors in the surgical ward per center per year. If we assume that at least one third of these are frail elderly this results in 10 eligible frail patients every month. With an inclusion rate of around 90% we should be able to include 108 patients per year and the duration of accrual will be 30 months. This entails an inclusion rate of 3-4 patients per center per month.

# 6. TREATMENT OF SUBJECTS

## 6.1 Investigational treatment

The intervention entails participation of a geriatric nurse and geriatrician in the perioperative treatment of the oncogeriatric surgical patient.

The geriatrician will see the patient preoperatively and pay attention to the following points:

1) General

diabetes
hypertension, angina pectoris, hartfealure, hartkleplijden
COPD

medication ( ascal, etc)
"accidental findings"

2) Geriatric

"frailty": GFI, ADL, i-ADL

D(epressie) D(ementie)D(elier): GDS, MMSE, DOS
visus, hearing
(in) mobility
neurological disease (M. Parkinson)
weight loss/ malnutrition
prostatism/incontinence
estimation quality of life/social context

"oncological statistical prognosis/life expectancy"

Based on the preoperative findings the geriatric team will plan measures to prevent adverse effects. These may include dietary measures, changes in medication and medication to prevent delirium. In the postoperative period this will include close daily monitoring of the frail patients and when necessary adaptation of their treatment and care plans. Multi-component interventions to achieve best-supportive care in individual treatment plans will be implemented. These will be focused on electrolyte- and fluid levels, pain management, pharmacological clearance, miction and defecation, nutrition, early mobilization and rehabilitation, sleep, vision, hearing and cognition. Any change in treatment will occur in collaboration with the physician responsible for daily care on the surgical ward. The geriatrician will be the supervisor of the geriatric team. The Delirium observation scale (DOS) will be used to screen for delirium by the nurse (Schuurmans 2003) and the Delirium Rating Scale (DRS) will be used to measure the severity of the delirium (Trzepazc 1998, 2001). To ensure uniformity of geriatric intervention in participating centres a daily checklist will be used (adapted from Kalisvaart 2005). Every item on this checklist will have to be addressed and checked by the member of the geriatric team involved with the patient at that time.

Geriatric care will not be withheld from patients in the standard care group. Should the treating physician see fit to ask for a consultation from a geriatrician this will be done as usual. This is no reason to withdraw patients from the study as consultation by a geriatrician at any moment is part of standard care.

**Checklist geriatrisch team**

Punten van aandacht:

Oriëntatie

Mobiliteit

Vijf zintuigen

Pijn

Slaap

Intake

Familie

Opvolgend advies

□= aankruisen indien verricht/aanwezig/van toepassing

D= dagelijks controleren/verrichten

A= punt van aandacht

**Oriëntatie**

□ Bekende objecten van thuis aanwezig

□ Bed aan raamzijde /hoek

D: kort gesprek, indien noodzakelijk jezelf voorstellen, geef informatie

A: is de kamer te druk voor deze patiënt (1-2 personen per kamer)

**Cognitieve problemen**

□ Kalender

□ Klok met zichtbare nummers of eigen klok

□ Nachtlampje aan, indien niet mogelijk vraag de familie naar een nachtlampje

D: gesprek mbt oriëntatie (wie,wat,waarom,waar)

A: verpleeegkundige vertelt wie ze is, waarom ze er is en wat ze doet

**Mobiliteit**

D:verwijder CAD/infuus/drain indien mogelijk

D: loopt de patiënt rond?

□ Ja geen actie □ Nee □ Fysiotherapie ingeschakeld

Zit de patiënt

□ op stoel, gezicht naar raam of gang

□ op stoel, buiten eigen kamer

Kan de patiënt het toilet op tijd bereiken?

□ is het urinaal/postoel binnen handbereik?

□ bel binnen handbereik (weet de patiënt hoe deze te gebruiken)

**Zintuigen**

*Zien*

Heeft patiënt een bril?

□ Ja □ Nee

Is de bril van patiënt aanwezig?

□ leesbril

□ andere bril

Indien slecht zicht ondanks correctie

□ gesproken boeken

□ drank niet in glazen maar drinkbeker

A:observeer, benader de patiënt van de beste zijde

*Horen*

Heeft de patiënt moeite met het verstaan van gesproken woorden?

□ Nee (geen actie)

□ Ja □ gehoorapparaat aanwezig en in

A: Gebruik apparaat gedurende de dag

Begin het gesprek

Kijk de patiënt aan tijdens gesprek

Spreek rustig

Benader patiënt van beste zijde

**Pijn**

Heeft de patiënt pijn?

□ Nee (geen actie)

□ Ja

Pijnmedicatie gestart □ Ja □ Nee

A : Observeer de pijn, ernst en wanneer deze optreedt

Schakel een arts in indien noodzakelijk

Besteed aandacht aan de bijweringen van de analgetica

**Slaap**

Slaapt de patiënt goed?

□ Ja (geen actie)

□ Nee

A: Indien mogelijk meer activiteiten aanbieden gedurende de dag

Sederende medicatie? Vraag arts deze te veranderen indien noodzakelijk

Slaap medicatie. Vraag arts deze te stoppen of veranderen

Haal patiënt overdag uit bed

Activiteitenprogramma

Max 1 uur middagslaap

Indien sprake van angst/probleem mbt slaap-> ritueel voor het slapen gaan

Indien kamer te levendig evt andere kamer

Slaapmedicatie noodzakelijk?

**Intake**

*Vloeibaar*

Patiënt drinkt alles wat hij/zij krijgt

□ Ja (geen actie) □ Nee (Stimuleer en bepaal ureum/creatinine na 2 dagen om hydratie te beoordelen)

*Vast voedsel*

Was de patiënt de afgelopen 5 dagen thuis een slechte eter?

□Ja (schakel diëtiste in) □Nee (geen actie)

Smaakt het eten goed?

□Ja (geen actie) □Nee (extra stimulatie)

D: defaecatie patroon, indien na 2 dagen geen defaecatie informeer arts

Eet alles

□Ja (geen actie) □Nee (schakel diëtiste in, overweeg tussendoortjes)

**Familie**

Indien pt verward kan de verpleegkundige de familie vragen

Vaker op bezoek te komen

Krant mee te nemen en evt voor te lezen

Praat over het hier en nu, thuis

**Medicatielijst**

Voorgeschreven medicatie gecontroleerd □Ja □Nee

Veranderingen aangebracht □Ja □Nee

Veranderingen + reden:

**Lab**

Lab uitslagen bekeken □Ja □Nee □n.v.t.

Extra lab aangevraagd □Ja □Nee

Reden:

**Advies**

Wordt advies van het team opgevolgd

□Ja (geen actie)

□Nee Goede reden-> geen actie

Twijfelachtig ->overleg met verpleegkundig team

# 7. METHODS

## 7.1 Study parameters/endpoints

### 7.1.1 Main study parameter/endpoint

The cumulative incidence of delirium (measured with the Delirium Observation Scale and the DSM IV criteria) up to 10 days postoperatively.

### 7.1.2 Secondary study parameters/endpoints

- Returning to the pre-operative living situation within 3 months postoperatively
- The Physical Component Summary measure (PCS) of the SF-36
- The Mental Component Summary measure (MCS) of the SF-36
- Complications during hospital stay including mortality
- Care Dependence Scale at discharge
- Direct health care and non-health care costs will be used as economic indicators.

## 7.2 Randomisation, blinding and treatment allocation

Minimisation will be used to randomize the patients.

Patients will be balanced per centre

Patients will be stratified according to:

- tumor location in chest (lung)or abdomen (liver, gastro-intestinal and pancreas) vs other tumors (breast, sarcoma, melanoma, thyroid)

## 7.3 Study procedures

In the outpatient clinic or a minimum of 24 hours prior to surgery the Groningen Frail Index Questionnaire will be applied to all patients over 65 years of age eligible for inclusion into the study. Application of the Groningen Frail Index Questionnaire is not of normal care in the participating centers. A research nurse will thus first ask the patient for informed consent and then apply the questionnaire. When a frailty index of >3 is found patients will be included in the study.

After obtaining informed consent the patient data will be filled in on the data form. These will include tumor type, surgery planned, age and sex. Then baseline values for the quality of life endpoints are obtained eg the Physical Component and Mental Component Summary Measure of the SF-36 and the Care Dependency scale. All these data are collected by a nurse. Preoperative Hb will be determined in the routine blood sample and no extra venapuncture is necessary.

## During treatment

## Daily screening for delirium in all patients up to discharge or 10 days postoperative with the delirium observation scale. On the first postoperative day the SF-36 score will also be measured. Recording of these data is not part of the usual treatment of patients and is part of the study protocol for both the intervention and the standard care group. Patients in the study group may receive changes in their diet or medication as the geriatric intervention team sees fit but no experimental drugs or investigational procedures will be used. A specialized nurse who is part of the geriatric team will daily go through the checklist for patients included in the intervention group. After referral with the geriatrician changes in treatment of these patients may be made. The changes made in the diet and medication of the patients depend on their medical situation and although the points of attention are standardized, the exact type of medication given or the dose can not be foreseen per individual patient.The patient with a positive screen on the DOS in the intervention group will be seen by geriatricians or consultant psychiatrists who are not participating in the geriatric liaison-intervention team in order to apply the DSM-IV criteria for the diagnosis of delirium.(American Psychiatric Association).

During hospital stay:

Daily recording of:

- Diagnostic procedures (including X-ray, CT-scan)
- Antibiotics prescribed
- Drugs prescribed
- Consultation by medical specialist
- Complications:
  - Wound infection
  - Anastomotic leakage (when applicable)
  - respiratory complications (pneumonia, combined clinical and radiological diagnosis)
  - cardio vascular complications events including arrythmia and cardiac failure
  - neurological events (CVA, RIND)
  - renal failure (as defined by a need for hemodialysis)
  - urinary infection
  - Thrombembolic events
  - pressure sores
  - death.

Total duration of hospital stay will be recorded as will days spent in the ICU when applicable

**At discharge:**

The Physical Component Summary measure (PCS) of the SF-36

The Mental Component Summary measure (MCS) of the SF-36

Care Dependency Scale

## Recording of these data is not part of the usual treatment of patients and is part of the study protocol for both the intervention and the standard care group.

## After the end of treatment (Follow-up):

## 3 months postoperatively the following data will be recorded. Recording of these data is not part of the usual treatment of patients and is part of the study protocol for both the intervention and the standard care group.

Return to pre-operative living situation

The Physical Component Summary measure (PCS) of the SF-36

The Mental Component Summary measure (MCS) of the SF-36

DATA COLLECTION SCHEME UNDER NORMAL CIRCUMSTANCES (IE NOT IN STUDY)

|  | At inclusion (outpatietnt or ward) | At admission | First post-operative day | Daily postoperatively | At Discharge |
| --- | --- | --- | --- | --- | --- |
| Groningen Frailty Index (ONLY IN LEIDEN) | X |  |  |  |  |
| Patient history (including smoking, drinking) | X |  |  |  |  |
| Living situation | X |  |  |  |  |
| Medication used | X | X | X | X | X |
| Packed cells given in 24 hours |  |  | X | X |  |
| Curative or palliative intent of operation? |  |  | X |  |  |
| Lab results (electrolytes, Hb, creatinin, leucocytes) | X |  | X | X |  |
| Diagnostic procedures |  |  | X | X |  |
| Days spent in the ICU |  |  | X | X |  |
| Comlpications |  |  | X | X |  |
| Consultation by specialist |  |  | X | X |  |

DATA COLLECTION SCHEME STANDARD CARE GROUP

|  | At inclusion (outpatietnt or ward) | At admission | First post-operative day | Daily postoperatively | At Discharge | 3 months postoperatively |
| --- | --- | --- | --- | --- | --- | --- |
| Groningen Frailty Index | X |  |  |  |  |  |
| Patient history (including smoking, drinking) | X |  |  |  |  |  |
| Living situation | X |  |  |  |  | X |
| Medication used | X | X | X | X | X | X |
| SF-36 |  | X | X |  | X | X |
| DOS |  | X | X | X |  |  |
| Packed cells given in 24 hours |  |  | X | X |  |  |
| Curative or palliative intent of operation? |  |  | X |  |  |  |
| Lab results (electrolytes, Hb, creatinin, leucocytes) | X |  | X | X |  |  |
| Diagnostic procedures |  |  | X | X |  |  |
| Days spent in the ICU |  |  | X | X |  |  |
| CDS | X |  |  |  | X |  |
| Comlpications |  |  | X | X |  |  |
| Consultation by specialist |  |  | X | X |  |  |

DATA COLLECTION SCHEME INTERVENTION GROUP

|  | At inclusion (outpatietnt or ward) | At admission | First post-operative day | Daily postoperatively | At Discharge | 3 months postoperatively |
| --- | --- | --- | --- | --- | --- | --- |
| Groningen Frailty Index | X |  |  |  |  |  |
| Patient history (including smoking, drinking) | X |  |  |  |  |  |
| Living situation | X |  |  |  |  | X |
| Medication used | X | X | X | X | X | X |
| Geriatric checklist |  | X | X | X |  |  |
| SF-36 |  | X | X |  | X | X |
| DOS |  | X | X | X |  |  |
| Packed cells given in 24 hours |  |  | X | X |  |  |
| Curative or palliative intent of operation? |  |  | X |  |  |  |
| Lab results (electrolytes, Hb, creatinin, leucocytes) | X |  | X | X |  |  |
| Diagnostic procedures |  |  | X | X |  |  |
| Days spent in the ICU |  |  | X | X |  |  |
| CDS | X |  |  |  | X |  |
| Complications |  |  | X | X |  |  |
| Consultation by specialist |  |  | X | X |  |  |

See appendix for exact content of tests

## 7.4 Withdrawal of individual subjects

Subjects can leave the study at any time for any reason if they wish to do so without any consequences. The investigator can decide to withdraw a subject from the study for urgent medical reasons.

## 7.5 Replacement of individual subjects after withdrawal

Individual subjects will not be replaced after withdrawal.

## 7.6 Follow-up of subjects withdrawn from treatment

Subjects withdrawn from treatment will receive standard care. Although we will not subject them to any tests, certain data such as length of hospital stay, complications and living situation 3 months postoperative will be recorded.

# 8. SAFETY REPORTING

## 8.1 Section 10 WMO event

In accordance to section 10, subsection 1, of the WMO, the investigator will inform the subjects and the reviewing accredited METC if anything occurs, on the basis of which it appears that the disadvantages of participation may be significantly greater than was foreseen in the research proposal. The study will be suspended pending further review by the accredited METC, except insofar as suspension would jeopardise the subjects’ health. The investigator will take care that all subjects are kept informed.

## 8.2 Adverse and serious adverse events

Adverse events are defined as any undesirable experience occurring to a subject during a clinical trial, whether or not considered related to the investigational drug. All adverse events reported spontaneously by the subject or observed by the investiga­tor or his staff will be recorded. No adverse effects of geriatric intervention are known to us.

A serious adverse event is any untoward medical occurrence or effect that at any dose results in death;

- is life threatening (at the time of the event);
- requires hospitalisation or prolongation of existing inpatients’ hospitalisation;
- results in persistent or significant disability or incapacity;
- is a congenital anomaly or birth defect;
- is a new event of the trial likely to affect the safety of the subjects, such as an unexpected outcome of an adverse reaction, lack of efficacy of an IMP used for the treatment of a life threatening disease, major safety finding from a newly completed animal study, etc.

All SAEs will be reported to the accredited METC that approved the protocol, according to the requirements of that METC.

## 8.3 Follow-up of adverse events

All adverse events will be followed until they have abated, or until a stable situation has been reached. Depending on the event, follow up may require additional tests or medical procedures as indicated, and/or referral to the general physician or a medical specialist.

## 8.4 Data Safety Monitoring Board (DSMB)

An independent data monitoring committee will perform the interim analysis and decide whether the trial should be stopped. All members of this committee should be excluded from the trial. The statistical analysis will not be disclosed to anyone who is not a member of this committee

# 9. STATISTICAL ANALYSIS

## 9.1 Descriptive statistics

The primary endpoint occurrence of delirium within 10 days after surgery will be analyzed using the Chi-square test. Delirium will be presented as qualitative data. The secondary endpoints will be analyzed using a student-T test. These analyses will be two sided.

Further analyses will be performed by logistic regression using the occurrence of delirium as dependent variable.

# 10. ETHICAL CONSIDERATIONS

## 10.1 Regulation statement

All patients will be informed of the aims of the study, the procedures and the mechanism of treatment allocation. They will be informed as to the strict confidentiality of their patient data, but that their medical records may be reviewed for trial purposes by authorized individuals other than their treating physician.

It will be emphasized that the participation is voluntary and that the patient is allowed to refuse further participation in the protocol whenever he/she wants. This will not prejudice the patient’s subsequent care. Documented informed consent must be obtained for all patients included in the study before they are registered in the study.

This study is conducted in agreement with the Declaration of Helsinki (Tokyo, Venice, Hong Kong, Somerset West and Edinburgh amendments) and in accordance with the Medical Research Involving Human Subjects Act (WMO) and other guidelines, regulations and Acts

According to the ICH guidelines on Good Clinical Practice and Dutch law, the written informed consent form should be signed and personally dated by the patient and the responsible investigator.

## 10.2 Recruitment and consent

Patients will be informed on the study by a research nurse at the outpatient clinic or 24 hours prior to surgery in case of “acute” admissions. The research nurse will give the patient the information letter and they will be given up to 24 hours prior to surgery to consider their decision. Upon consent the patient and the research nurse will sign the informed consent form. The patient information letter and informed consent form are attached as a separate document.

## 10.3 Benefits and risks assessment, group relatedness

Delirium is a highly prevalent and potential lethal postoperative complication in elderly patients. The literature suggests that perioperative intervention by a geriatric team decreases the incidence of this complication. The intervention by the geriatric team entails adjustments in the daily routine of the patient, the diet and medication prescribed but no additional invasive of uncomfortable procedures. The intervention is tailored to make the patient as comfortable as possible. We therefore think it is beneficial to expose the participating subjects to extra questionnaires perioperatively that will take up some of their time as this is the only way for us to show the benefit of the geriatric intervention. This study has to be performed in the fragile elderly patients as they are the patients most likely to develop postoperative delirium and most likely to benefit from the intervention.

There are no risks known to us related to a geriatric intervention or the answering of questionnaires.

## 10.4 Compensation for injury

The investigators wish to obtain dispensation from the insurance. This has been requested from the METC.

# 11ADMINISTRATIVE ASPECTS AND PUBLICATION

## 11.1 Handling and storage of data and documents

All data will be handled confidentially and anonymously. A subject identification code list will be used to link the data to the subject. The code will not be based on the patients intials and birth-date. The code will consist of a number for the hospital the patient was treated in and a serial number according to time of entering the study. The key to the code will be safeguarded by the investigator. The handling of personal data will comply with the Dutch Personal Data Protection Act.

## 11.2 Amendments

All amendments will be notified to the METC that gave a favourable opinion.

## 11.3 Annual progress report

The sponsor/investigator will submit a summary of the progress of the trial to the accredited METC once a year. Information will be provided on the date of inclusion of the first subject, numbers of subjects included and numbers of subjects that have completed the trial, serious adverse events/ serious adverse reactions, other problems, and amendments.

## 11.4 End of study report

The investigator [sponsor] will notify the accredited METC [and the competent authority] of the end of the study within a period of 8 weeks [90 days]. The end of the study is defined as 3 months postoperative for the last patient included.

In case the study is ended prematurely, the investigator [sponsor] will notify the accredited METC [and the competent authority within 15 days], including the reasons for the premature termination.

 Within one year after the end of the study, the investigator/sponsor will submit a final study report with the results of the study, including any publications/abstracts of the study, to the accredited METC [and the Competent Authority].

## 11.5 Public disclosure and publication policy

The research data will be publicly disclosed and published independent of the outcome of this study. Members of the writing committee including the principal investigators, will all participate in the publication process.

# 12. REFERENCES

- Audisio RA, Bozzetti F, et al. The surgical management of elderly cancer patients recommendations of the SIOG surgical task force. Eur J Cancer, 2004; 40: 926-38
- Brennan T.A., Leape, L.L., Laird, N.M., et al). Incidence of adverse events and negligence in
- hospitalized patients: Results of the Harvard Medical Practice Study. N Engl J Med, 1991;324, 370-376.
- Damhuis R.A.M, Meurs C.J.C, Meijer W. Postoperative mortality after cancer surgery in octogenarians and nonagenarians; results from a series of 5,390 patients. World J Surg Oncol, 2005; 3:71.
- Dijkstra et al. Het meten van zorgafhankelijkheid met de Zorgafhankelijkheidsschaal (ZAS): een handleiding. 1999. Groningen. Noordelijk Centrum voor Gezondheidsvraagstukken. ISBN 90-72156-46-3.
- Fentiman I.S, Are the elderly receiving appropriate therapy for cancer?. Ann. Oncol. 1996; 7: 657-58
- Francis J., Martin, D., Kapoor, W.N. A prospective study of delirium in hospitalized elderly. JAMA, 1990;263, p. 1097-1101.
- Franco K., Litaker D., Locala J. Bronson D. The Cost of delirium in the Surgical Patient.
- Psychosomatics, 2001; 42:1, 68-73.
- Frieswijk N., Buunk A.P., Steverink N., Slaets J.P.J. The interpretation of Social Comparison and Its Relation to Life Satisfaction Among Elderly People: Does Frailty Make a Difference? J Gerontol B Psychol Sci Soc Sci ,2004; 59B;5:P250-P257.
- Hoekstra HJ. Cancer surgery in the elderly Eur J Cancer. 2001; 37 (7):235-44
- Inouye S.K. Bogardus S.T., Charpentier P.A,, Leo-Summers L,Campora D.A, Holford T.A, Cooney L.M. A multicomponent intervention to prevent delirium in hospitalized older patients. N Engl J Med , 1999; 340; 9, 669-676
- Kalisvaart K.J., de Jonghe JF, Bogaards MJ, Vreeswijk R, Egberts TC, Burger BJ, Eikelenboom P, van Gool WA. Haloperidol prophylaxis for elderly hip-surgery patients at risk for delirium: a randomized placebo-controlled study. J Am Geriatr Soc, 2005;53(10):1658-66.
- Kalisvaart. Primary prevention of delirium in the elderly. Thesis 2005
- Marcantonio ER, Flacker JM, Wright RJ, Resnick NM.Reducing delirium after hip fracture: a randomized trial.J Am Geriatr Soc. 2001 May;49(5):516-22.
- Mast van der R.C., Huyse FJ, Drooglever H.A. et al. Dutch Institute for Healthcare Improvement (CBO) Guideline Delirium. 2004, Boom Amsterdam..
- Olde Rikkert, M. In: Notitie Werkgroep Ouderenzorg t.b.v. ZonMw deelprogramma Klinische Geriatrie. Ouderen nu en in de toekomst. Gezondheid, verpleging en verzorging 2000-2020. 2004; RIVM 270502001.
- Repetto L., Ventrino A, Fratino L. et al. Geriatric oncology: a clinical approach to the older patient with cancer. Eur J Cancer, 2003; 39 (7): 870-880.
- Rothschild, J.M., Bates, D.W., Leape, L.L. Preventable medical injuries in older patients. Arch Intern Med, 2000; 160, 2717-2728.
- Sager, M.A., Rudberg, M.A., Jalaluddin, M. et al.). Promoting well-being in frail elderly people: Theory and intervention.Dissertation, 2004, Groningen University.
- Schuurmans MJ, Shortridge-Bagget LM, Duursma SA. The Delirium Observation Screening Scale: a screening instrument for delirium. Res Theory Nurs Pract. 2003 17(1):31-50
- Schuurmans, H., Steverink, N., Lindenberg, S., Frieswijk, N., Slaets, J.P.J.. Old or frail: what tells us more? J Gerontol A Biol Sci Med Sci. 2004 Sep;59(9):M962-5.
- Slaets JP. [The importance of the 'frailty-concept' in geriatric assessment]. Tijdschr.Gerontol.Geriatr.1998;29:276-8.
- Slaets JPJ. De oudere patiënt. In Consultatieve inwendige geneeskunde. ROB Gans, SJ Hoorntje en RJM Strack van Schijndel (red). Bohn Stafleu van Loghum, Houten 2004.
- Slaets, J.P.J., Kauffmann, R.H., Duivenvoorden, H.J., Pelemans, W., Schudel, W.J.). A randomized trial of geriatric liaison intervention in elderly medical inpatients. Psychosom Med. 1997 Nov-Dec;59(6):585-91.
- Steverink, N., Slaets, J.P.J., Schuurmans, H., Lis, M. van. Measuring frailty: development and testing of the Groningen Frailty Indicator (GFI). Gerontologist, 2001; 41SI, 236-237.
- Trzepazc P.T., Mulsant, B.H.,Amanda-Dew, M., Pasternak, R.,Sweet, R.A. et al. Is delirium different when it occurs in dementia? A study using the delirium rating scale. J Neuropsychiatry Clin Neurosci. 1998 Spring;10(2):199-204.
- Van Hemert A.M., Van der Mast RC, Hengeveld MW et al. Excess mortality in general hospital patients with delirium: a 5 year follow up of 519 patients seen in psychiatric consultation. J Psychosom Res, 1994;38: 339-346.
- Vercelli M, Capocaccia D, Quaglia A, Casella C, Puppo A, Coebergh J W W and EUROCARE Working Group. Relative survival in elderly European cancer patients: evidence for health care inequalities. Crit Rev Oncol Hematol, 2000; 35: 161-79
- Wymenga AN, Slaets JP, Sleijfer DT. Treatment of cancer in old age, shortcomings and challenges. Neth.J.Med. 2001;59:259-66

Informed consent. Patiënten-informatiebrief behorend bij

**Onderzoek naar postoperatief functioneren van kwetsbare ouderen**

Geachte heer/mevrouw,

Binnenkort ondergaat u een operatie.

U zult rondom deze operatie enkele dagen tot weken opgenomen zijn op de afdeling chirurgie of oncologie van dit ziekenhuis. Wij onderzoeken op dit moment in dit ziekenhuis hoe patiënten ouder dan 65 jaar herstellen van een operatie. Wij zijn daarbij voornamelijk geïnteresseerd in het beloop na de operatie en in hun functioneren in de eerste 3 maanden na de operatie. Wij onderzoeken mogelijkheden om de zorg rondom de operatie te verbeteren en daarmee het beloop na de operatie zo gunstig mogelijk te laten verlopen. Met dit onderzoek verwachten we aan te tonen dat met de medebehandeling door een team gespecialiseerd in de ouderengeneeskunde mogelijke problemen sneller gesignaleerd worden en het herstel optimaler zal verlopen . Wij willen u daarom vragen deel te nemen aan dit onderzoek.

Als u besluit aan dit onderzoek deel te nemen houdt dit voor u het volgende in:

Een verpleegkundige zal u een aantal vragen stellen met betrekking tot uw gezondheid en

woonomstandigheden op dit moment. Dit neemt ongeveer 15 minuten in beslag. Aan de hand van de uitkomsten zal een inschatting worden gemaakt hoe groot de kans is dat de operatie en de ziekenhuisopname van invloed kunnen zijn op uw functioneren in de eerste 3 maanden na de operatie. Dit bepaalt mede of u deel kunt nemen aan dit onderzoek.

Het is mogelijk dat bij deelname aan dit onderzoek een geriater (internist gespecialiseerd in de zorg van de oudere patiënt) en verpleegkundig specialist betrokken wordt bij uw behandeling tijdens uw opname in het ziekenhuis. Het al dan niet betrekken van deze mensen bij uw behandeling wordt door het loting bepaald. De kans dat een geriater en verpleegkundig specialist bij uw behandeling betrokken zullen worden is 50%. De geriater zal u in dat geval voorafgaand aan de operatie onderzoeken en eventueel veranderingen in uw medicatie of dieet aanbrengen. Na de operatie komt een verpleegkundig specialist dagelijks bij u langs om te kijken hoe het gaat. Zij zal in overleg met de geriater en de artsen die u behandelen waar nodig uw behandeling veranderen c.q. verbeteren. Aan alle patiënten die meedoen aan het onderzoek zullen tijdens de opname dagelijks enkele vragen worden gesteld met betrekking tot het functioneren en het welbevinden op dat moment. Dat neemt niet meer dan enkele minuten van uw tijd in beslag.

Bij ontslag uit het ziekenhuis en 3 maanden na de operatie zullen dezelfde vragen met

betrekking tot gezondheid, functioneren en woonomstandigheden opnieuw worden gesteld. Het beantwoorden van deze vragen zal ongeveer 30 tot 60 minuten van uw tijd vergen.

Bij het onderzoek zullen gegevens uit uw medisch dossier worden gebruikt. Het gaat hier om uw medische voorgeschiedenis, medicatie , gegevens betreffende uw operatie, en het beloop na uw operatie inclusief uitslagen van bloedonderzoek, en uitslagen van evt aanvullende onderzoeken (rontgen) die na de operatie bij u worden uitgevoerd. Artsen, verpleegkundigen en onderzoekers betrokken bij dit onderzoek zullen toegang hebben tot deze gegevens. Bij de analyse van deze gegevens blijft uw privacy gewaarborgd.

Aan deelname zijn geen risico’s verbonden en uw operatieve behandeling wordt hierdoor niet beïnvloed. Het staat u op ieder ogenblik vrij om deelname aan dit onderzoek te weigeren. Dit heeft geen consequenties voor uw verdere behandeling. Ook bent u volledig vrij om zonder opgave van redenen op ieder moment uw deelname aan het onderzoek te beëindigen. Ook dit heeft geen enkel effect op de wijze waarop wij u verder zullen behandelen. Mocht u tijdens deelname aan dit onderzoek behandeling van een geriater nodig hebben dan zal u die ontvangen onafhankelijk van de groep waarin u door loting terecht bent gekomen. Deelname aan het onderzoek wordt hierdoor niet beïnvloed.

Nu deelname aan dit wetenschappelijk onderzoek naar zijn aard voor u geen risico met zich meebrengt, heeft de Medisch Ethische Toetsingscommissie van het UMCG aan de onderzoekers ontheffing verleend van de verplichting om een verzekering af te sluiten.

Voor informatie kan u in eerste instantie contact opnemen met: J. Slaets tel: 050-3612943

Indien u los van de onderzoekers informatie of advies wenst kan u contact opnemen met een onafhankelijke arts (niet betrokken bij dit onderzoek): Dr. J.T.M. Plukker, afdeling Heelkunde, UMCG, tel: 050-3616161

Bij voorbaat dank voor uw medewerking,

Namens het onderzoeksteam

Prof. Dr. J.P.J. Slaets

Hoofd Academisch Centrum Ouderengeneeskunde

UMCG

Toestemmingsformulier behorend bij

**Onderzoek naar postoperatief functioneren van kwetsbare ouderen**

Ik stem erin toe deel te nemen aan bovengenoemd onderzoek. Door loting wordt bepaald of een geriater en verpleegkundig specialist betrokken worden bij mijn behandeling tijdens de ziekenhuisopname.

Tijdens dit onderzoek zal ik een aantal keren vragen beantwoorden met betrekking tot mijn gezondheid, functioneren en woonomstandigheden. De mensen betrokken bij dit onderzoek (artsen, verpleegkundigen, onderzoekers) hebben toegang tot mijn medisch dossier. Resultaten van dit onderzoek zullen worden gepubliceerd in medische tijdschriften waarbij mijn privacy gewaarborgd blijft.

Aan deelname zijn geen risico’s verbonden en mijn operatieve behandeling wordt hierdoor niet beïnvloed. Het staat mij op ieder ogenblik vrij om deelname aan dit onderzoek te weigeren. Dit heeft geen consequenties voor verdere behandeling. Ook ben ik volledig vrij om zonder opgave van redenen op ieder moment deelname aan het onderzoek te beëindigen. Ook dit heeft geen enkel effect op de wijze waarop ik verder zal worden behandeld.

Handtekening Patiënt Handtekening lid onderzoeksteam

Naam Patiënt Naam lid onderzoeksteam

Datum:

Ziekenhuis

**Groningen Frailty Index**

**Mobiliteit**

Kan patiënt zonder enige hulp van iemand anders zelfstandig deze taak uitvoeren? (gebruik maken van hulpmiddelen als stok, rolator, rolstoel, geldt als zelfstandig

1. Boodschappen doen JA/NEE

2. Buitenshuis rondlopen (rondom huis of naar de buren) JA/NEE

3. Aan- en uitkleden JA/NEE

4. Toiletbezoek JA/NEE

**Lichamelijke fitheid**

5. Welk rapportcijfer geeft patiënt zichzelf voor lichamelijke fitheid? (0-10, 0 is erg slecht, 10 is erg goed)

**Visus**

6. Ondervindt patiënt problemen in dagelijks leven door slecht zien? JA/NEE

**Gehoor**

7. Ondervindt patiënt problemen in het dagelijks leven door slecht horen? JA/NEE

**Voeding**

8. Is patiënt afgelopen 6 maanden veel (6kg) afgevallen zonder dit zelf te willen? (of 3 kg in een maand) JA/NEE

**Co-morbiditeit**

9. Gebruikt patiënt momenteel 4 of meer verschillende soorten medicijnen? JA/NEE

**Cognitie**

10. Heeft patiënt momenteel klachten over zijn geheugen (of bekend met dementie) JA/NEE

**Psychosociaal**

11. Ervaart patiënt wel eens een leegte om zich heen? JA/NEE

12. Mist patiënt wel eens mensen om zich heen? JA/NEE

13. Voelt patiënt zich wel eens in de steek gelaten? JA/NEE

14. Heeft patiënt zich de laatste tijd somber of neerslachtig gevoeld? JA/NEE

15. Heeft patiënt zich de laatste tijd nerveus of angstig gevoeld? JA/NEE

**Scoring GFI**:

Vraag 1t/m 4: zelfstandig = 0, niet zelfstandig = 1

Vraag 5: 0-6= 1, 7-10= 0

Vraag 6 t/m 9; nee = 0, ja = 1

Vraag 10: nee = 0, soms = 0, ja = 1

Vraag 11 t/m 15 nee= 0, soms = 1, ja= 1

**CARE DEPENDENCY SCORE**

**CDS**-vpl *1*

Beoordeel de patiënt, die aan uw zorg is toevertrouwd, op elk van de 15 kenmerken van de

zorgafhankelijkheidsschaal. Geef aan welke beschrijving het beste van toepassing is. Ga uit van

de mogelijkheden die de patiënt bezit en niet zozeer welke handelingen de verzorgende of verpleegkundige overneemt.

Voorbeeld:

**1 Eten en drinken De mate waarin de aan uw zorg toevertrouwde patiënt in staat is zelfstandig te voldoen aan de behoefte aan eten en drinken**

*1 Volledig zorgafhankelijk*

*2 In grote mate zorgafhankelijk*

*3 Gedeeltelijk zorgafhankelijk*

*4 Beperkt zorgafhankelijk*

*5 Vrijwel zelfstandig*

Geef van alle 15 kenmerken een beoordeling, sla geen kenmerk over. Ten slotte wordt u gevraagd in vraag 16 aan te geven welke omschrijving van zorgafhankelijkheid van toepassing is op de patiënt als geheel. Probeer bij twijfel tussen 2 beschrijvingen tot één keuze te komen, door in te schatten welke mogelijkheid de betreffende patiënt bezit.

Er zijn 15 kenmerken met ieder 5 criteria van zorgafhankelijkheid opgenomen in de

zorgafhankelijkheidsschaal. OMCIRKEL het CIJFER van het criterium welke het beste bij de

betreffende patiënt past. (Maak uw keuze door één van de cijfers te omcirkelen)

**1 Eten en drinken De mate waarin de patiënt in staat is zelfstandig te voldoen aan de behoefte aan eten en drinken**

*1 Volledig zorgafhankelijk*

*2 In grote mate zorgafhankelijk*

*3 Gedeeltelijk zorgafhankelijk*

*4 Beperkt zorgafhankelijk*

*5 Vrijwel zelfstandig*

**2 Incontinentie De mate waarin de patiënt het vermogen heeft de uitscheiding van urine en faeces willekeurig te beheersen**

*1 Volledig zorgafhankelijk*

*2 In grote mate zorgafhankelijk*

*3 Gedeeltelijk zorgafhankelijk*

*4 Beperkt zorgafhankelijk*

*5 Vrijwel zelfstandig*

**3 Lichaamshouding De mate waarin de patiënt in staat is bij een bepaalde activiteit een juiste houding aan te nemen**

*1 Volledig zorgafhankelijk*

*2 In grote mate zorgafhankelijk*

*3 Gedeeltelijk zorgafhankelijk*

*4 Beperkt zorgafhankelijk*

*5 Vrijwel zelfstandig*

**4 Mobiliteit De mate waarin de patiënt fysiek in staat is zich zelfstandig voort te bewegen**

*1 Volledig zorgafhankelijk*

*2 In grote mate zorgafhankelijk*

*3 Gedeeltelijk zorgafhankelijk*

*4 Beperkt zorgafhankelijk*

*5 Vrijwel zelfstandig*

**5 Dag-nachtritme De aard van het slaap-waakpatroon (bio-ritme) van de patiënt**

*1 Volledig zorgafhankelijk*

*2 In grote mate zorgafhankelijk*

*3 Gedeeltelijk zorgafhankelijk*

*4 Beperkt zorgafhankelijk*

*5 Vrijwel zelfstandig*

**6 Aan- en uitkleden De mate waarin de patiënt beschikt over vaardigheden om zich zelfstandig aan- en uit te kleden**

*1 Volledig zorgafhankelijk*

*2 In grote mate zorgafhankelijk*

*3 Gedeeltelijk zorgafhankelijk*

*4 Beperkt zorgafhankelijk*

*5 Vrijwel zelfstandig*

**7 Lichaamstemperatuur De mate waarin de patiënt in staat is zelfstandig de lichaamstemperatuur te beschermen tegen externe invloeden**

*1 Volledig zorgafhankelijk*

*2 In grote mate zorgafhankelijk*

*3 Gedeeltelijk zorgafhankelijk*

*4 Beperkt zorgafhankelijk*

*5 Vrijwel zelfstandig*

**8 Hygiëne De mate waarin de patiënt in staat is zelfstandig zorg te dragen voor zijn/haar lichaamsverzorging**

*1 Volledig zorgafhankelijk*

*2 In grote mate zorgafhankelijk*

*3 Gedeeltelijk zorgafhankelijk*

*4 Beperkt zorgafhankelijk*

*5 Vrijwel zelfstandig*

**9 Vermijden van gevaar De mate waarin de patiënt in staat is zelfstandig voor zijn/haar eigen veiligheid te zorgen**

*1 Volledig zorgafhankelijk*

*2 In grote mate zorgafhankelijk*

*3 Gedeeltelijk zorgafhankelijk*

*4 Beperkt zorgafhankelijk*

*5 Vrijwel zelfstandig*

**10 Communicatie De mate waarin de patiënt in staat is te communiceren**

*1 Volledig zorgafhankelijk*

*2 In grote mate zorgafhankelijk*

*3 Gedeeltelijk zorgafhankelijk*

*4 Beperkt zorgafhankelijk*

*5 Vrijwel zelfstandig*

**11 Contact met anderen De mate waarin de patiënt in staat is tot het aangaan, onderhouden en afbreken van sociaal contact**

*1 Volledig zorgafhankelijk*

*2 In grote mate zorgafhankelijk*

*3 Gedeeltelijk zorgafhankelijk*

*4 Beperkt zorgafhankelijk*

*5 Vrijwel zelfstandig*

**12 Waarde- en normbesef De mate waarin de patiënt in staat is zelfstandig leefregels te hanteren**

*1 Volledig zorgafhankelijk*

*2 In grote mate zorgafhankelijk*

*3 Gedeeltelijk zorgafhankelijk*

*4 Beperkt zorgafhankelijk*

*5 Vrijwel zelfstandig*

**13 Dagelijkse activiteiten De mate waarin de patiënt in staat is zelfstandig invulling te geven aan de dagelijkse bezigheden binnen de woongroep**

*1 Volledig zorgafhankelijk*

*2 In grote mate zorgafhankelijk*

*3 Gedeeltelijk zorgafhankelijk*

*4 Beperkt zorgafhankelijk*

*5 Vrijwel zelfstandig*

**14 Recreatieve activiteiten De mate waarin de patiënt in staat is zelfstandig deel te nemen aan ontspannende activiteiten buiten de woongroep**

*1 Volledig zorgafhankelijk*

*2 In grote mate zorgafhankelijk*

*3 Gedeeltelijk zorgafhankelijk*

*4 Beperkt zorgafhankelijk*

*5 Vrijwel zelfstandig*

**15 Leervermogen De mate waarin de patiënt in staat is om zelfstandig kennis en/of vaardigheden aan te leren dan wel het geleerde in stand te houden**

*1 Volledig zorgafhankelijk*

*2 In grote mate zorgafhankelijk*

*3 Gedeeltelijk zorgafhankelijk*

*4 Beperkt zorgafhankelijk*

*5 Vrijwel zelfstandig*

**16 Samenvatting Geef aan welke omschrijving van zorgafhankelijkheid van toepassing is op de patiënt**

*1 Volledig zorgafhankelijk*

*2 In grote mate zorgafhankelijk*

*3 Gedeeltelijk zorgafhankelijk*

*4 Beperkt zorgafhankelijk*

*5 Vrijwel zelfstandig*

***DELIRIUM RATING SCALE-R-98***

***(DRS-R-9^S^)***

Vertaling: H.N. Sno, A.C. van der Mast.

Dit is een herziene versie van de Delirium Rating Scale (Trzepacz et al. 1988). Deze wordt gebruikt voor een eerste onderzoek en herhaalde metingen van de ernst van delirante symptomen. De som van de dertien itemscores is een ernstscore. **In** aanvulling op het psychiatrisch onderzoek van de patiënt worden alle be­schikbare bronnen van informatie gebruikt bij het scoren van de items (verpleegkundigen, familie, status). Bij een reeks herhaalde metingen van de ernst van het delirium, dienen tussen de metingen redelijke tijdsin­tervallen gekozen te worden om veranderingen van betekenis vast te leggen aangezien de ernst van de de­lirante symptomen zonder interventies kan wisselen.

DRS-R-98 Ernst-schaal

**1 Stoornis van het slaap-waakritme**

Maak bij het scoren van het slaap-waakpatroon gebruik van alle informatiebronnen, zoals van familie, hulp­verleners, verpleegkundige rapporten en de patiënt. Probeer een onderscheid te maken tussen slapen en rusten met gesloten ogen.

0 Niet aanwezig

1 Lichte verstoring van het 's nachts doorslapen of zo nu en dan slaperigheid overdag

2 Matig ernstige verstoring van het slaap-waakritme (bijvoorbeeld: *valt* in slaap tijdens gesprekken, doet overdag dutjes of wordt 's nachts meerdere malen kortdurend wakker, gepaard gaande met verwardheid/gedragsveranderingen of slaapt 's nachts heel weinig

3 Ernstige verstoring van slaap-waakritme (bijvoorbeeld omkering van het dag-nachtritme) of ernstige circadiane fragmentatie gepaard gaand met meerdere periodes van slapen en waken of ernstige slapeloosheid

**2 Waarnemingsstoornissen en hallucinaties**

Illusionaire vervalsingen en hallucinaties kunnen iedere zintuiglijke modaliteit betreffen. Waarnemingsstoor­nissen zijn 'eenvoudig' indien deze ongecompliceerd zijn, zoals geluid, lawaai, kleur, vlekken of flitsen en 'complex' wanneer deze multidimensioneel zijn, zoals stemmen, muziek, mensen, dieren of voorvallen. Scoor op basis van de anamnese van de patiënt, van de verzorger of van observatie.

0 Niet aanwezig

1 Lichte waarnemingsstoornissen (bijvoorbeeld gevoelens van derealisatie of depersonalisatie; of de patiënt is niet in staat om een onderscheid te maken tussen dromen en werkelijkheid)

2 Aanwezigheid van illusionaire vervalsingen

3 Aanwezigheid van hallucinaties

**3 Wanen**

Wanen kunnen ieder type zijn, maar betreffen meestal paranoïde wanen. Scoor op basis van anamnese van de patiënt, familie of verzorger. Scoor als waan indien het onwaarschijnlijk is dat de ideeën waar zijn, terwijl de patiënt erin gelooft en door logische argumenten niet op andere gedachten gebracht kan worden. Waan­ideeën kunnen niet anderszins verklaard worden door de culturele of religieuze achtergrond van de patiënt.

0 Niet aanwezig

1 Licht achterdochtig, zeer waakzaam of in beslag genomen

2 Ongewone en overwaardige ideeën die niet het niveau van een waan bereiken en waar zouden kunnen zijn

3 Wanen

**4 Affectlabiliteit**

Scoor het affect van de patiënt op basis van zichtbare uitingen van emoties en niet op basis van een beschrijving van wat de patiënt voelt.

0 Niet aanwezig

1 Het affect is enigszins veranderd of past niet helemaal bij de situatie; kan wisselen in verloop van uren: patiënten hebben emoties meestal onder controle

2 Het affect past vaak niet bij de situatie en kan wisselen in verloop van minuten; patiënten hebben meestal geen controle over emoties, hoewel ze wel reageren op correcties van anderen

3 Ernstige en aanhoudende emotionele ontremming; het affect wisselt snel, is niet passend bij de situatie en reageert niet op correcties van anderen

**5 Taal**

Scoor afwijkingen van gesproken, geschreven of gebarentaal, die niet toegeschreven kunnen worden aan dialect of stotteren. Let op tempo en intonatie, grammatica, begrip, semantische inhoud en benoemen. Test het begrip en benoemen zo nodig non-verbaal door de patiënt opdrachten te laten vervullen of te laten aan­wijzen.

0 Normaal taalgebruik

1 Lichte verslechtering zoals woordvindingsstoornissen of problemen met benoemen, tempo of intonatie.

2 Matig ernstige verslechtering zoals moeite met begrijpen of een tekortschietende zinvolle communicatie (semantische inhoud)

3 Ernstige verslechtering zoals betekenisloze semantische inhoud, woordsalade, mutisme of ernstig verminderd begrip

**6 Afwijkingen van het denkproces**

Baseer score van afwijkingen van denkprocessen op verbale en geschreven uitingen. Scoor dit item niet, in­dien een patiënt niet kan spreken of schrijven.

0 Normale denkprocessen

1 Tangentieel of circumstantieel

2 Verbanden zijn nu en dan onsamenhangend, maar merendeels te begrijpen

3 Verbanden zijn meestal onsamenhangend

**7 Motorische agitatie**

Scoor op basis van eigen observatie en die van anderen, zoals bezoek, familie en afdelingsstaf. Laat dyskinesie, tics of chorea buiten beschouwing.

0 Geen onrust of agitatie

1 Lichte onrust van grove motoriek of lichte plukkerigheid

2 Matig ernstige motorische agitatie, zoals duidelijke bewegingsonrust van de extremiteiten, rusteloos op en neer lopen, plukkerigheid, uittrekken van infuuslijnen enzovoort

3 Ernstige motorische agitatie, zoals motorische agressie of noodzaak van dwangmiddelen of separeren

**8 Motorische remming**

Scoor bewegingen op basis van directe observatie of observaties van anderen zoals familie, bezoek of af­delingsstaf. Laat remming als gevolg van parkinsonisme buiten beschouwing, evenals slaperigheid of slapen.

0 Geen traagheid van willekeurige bewegingen

1 Licht verminderde frequentie, spontaniteit of snelheid van de motoriek zodanig dat het enigszins interfereert met het onderzoek

2 Matige ernstig verminderde frequentie, spontaniteit of snelheid van motoriek zodanig dat het interfereert met deelname aan activiteiten of de zelfverzorging

3 Ernstige motorische remming met weinig spontane bewegingen

**9 Oriëntatie**

Aan patiënten die niet kunnen spreken kunnen visueel of auditief multiple-choice-antwoorden aangeboden worden. Patiënten die langer dan drie weken zijn opgenomen mogen er maximaal zeven dagen in plaats van twee dagen naast zitten. Desoriëntatie in persoon betekent het niet herkennen van bekende personen en is ongestoord bij het niet op de naam kunnen komen, terwijl er wel sprake is van herkenning. De meest ernstige vorm van desoriëntatie in persoon is het niet kennen van de eigen identiteit en is zeldzaam. Des­oriëntatie in persoon treedt meestal op na desoriëntatie in tijd enlof persoon.

0 Georiënteerd in persoon, plaats en tijd

1 Desoriëntatie in tijd (bijvoorbeeld meer dan twee dagen, of verkeerde maand of verkeerd jaar) of plaats (bijvoorbeeld gebouw, stad, provincie), maar niet beide

2 Desoriëntatie in tijd en plaats

3 Desoriëntatie in persoon

**10 Aandachtsconcentratie**

Bij patiënten met zintuiglijke beperkingen of die geïntubeerd zijn of hun handen niet kunnen gebruiken dient een andere modaliteit dan schrijven te worden getest. De aandachtsconcentratie kan onderzocht worden tij­dens het interview (bijvoorbeeld verbale perseveraties, afleidbaarheid en moeite met wisselen van de aan­dacht) enlof door het gebruiken van specifieke tests, bijvoorbeeld digit-scan.

0 Alert en oplettend

1 Licht afleidbaar of lichte moeite om de aandacht vast te houden, maar in staat zich te herstellen met behulp van aanwijzingen. Bij formeel testen worden slechts geringe fouten gemaakt en de patiënt reageert niet significant trager.

2 Matig ernstige aandachtsconcentratiestoornis met moeite aandacht te richten en vast te houden. Bij formeel testen maakt de patiënt verschillende fouten en heeft aansporing nodig om de aandacht te richten of de taak te voltooien.

3 Ernstige problemen met het richten enlof vasthouden van de aandacht, met veel onjuiste of incomplete antwoorden of onvermogen opdrachten op te volgen. Afleidbaar door andere geluiden of dingen die gebeuren in de omgeving.

**11 Kortetermijngeheugen**

Gedefinieerd als het herinneren van informatie (bijvoorbeeld drie items die ofwel verbaal of visueel worden aangeboden) na een interval van ongeveer twee tot drie minuten. Wanneer formeel getest wordt, dient de in­formatie eerst adequaat geregistreerd te zijn alvorens het geheugen getest wordt. Zowel het aantal pogingen om te registreren als het *effect* van het geven van een hint kan op het scoreformulier worden genoteerd. Het is patiënten niet toegestaan om oefenen gedurende de intervalperiode en zij gedurende die tijd worden afgeleid. De patiënt mag de onderzoeker pratend of non-verbaal de identiteit van de juiste items laten weten. Gebreken in het kortetermijngeheugen die opgevallen zijn tijdens het interview kunnen ook worden gebruikt.

0 Kortetermijngeheugen intact

1 Herinnert 2/3 items; zou zich het derde item herinneren nadat een aanwijzing betreffende de categorie is gegeven

2 Herinnert 1/3 items; zou zich andere items herinneren nadat aanwijzingen betreffende de categorie is gegeven

3 Herinnert 0/3 items

**12 Langetermijngeheugen**

Kan formeel onderzocht worden of door middel van een interview over herinnering van informatie omtrent persoonlijk verleden (bijvoorbeeld medische voorgeschiedenis of informatie of ervaringen die afgeleid kun­nen worden via een andere bron) of algemene informatie die cultureel relevant is. Wanneer formeel getest wordt, gebruik een verbale en/of visuele modaliteit voor drie items die adequaat geregistreerd en gerepro­duceerd worden na minimaal vijf minuten. De patiënt dient niet de gelegenheid te krijgen om te repeteren gedurende de intervalperiode bij een formele test.

Houd, wat betreft vragen omtrent algemene informatie, rekening met patiënten die de lagere school niet hebben afgemaakt of die mentaal geretardeerd zijn. Het scoren van de ernst van de gebreken kan een oor­deel inhouden over alle methoden waarop het langetermijngeheugen is onderzocht, inclusief de capaciteit van het langetermijngeheugen betreffende het recente en/of nabije verleden die gedurende het interview in­formeel is getest alsmede elke formele test van het langetermijngeheugen betreffende het recente verleden waarbij gebruik is gemaakt van drie items.

0 Geen significante stoornis van het langetermijngeheugen

1 Herinnert 2/3 items en/of heeft geringe moeite met het herinneren van details van andere langetermijn informatie

2 Herinnert 1/3 items en/of heeft matig ernstige moeite met het herinneren van andere langetermijninformatie

3 Herinnert 0/3 items en/of heeft zeer ernstige moeite met het herinneren van details van andere langetermijninformatie

**13 Visuospatieel oriëntatievermogen**

Onderzoek informeel en formeel. Let op de problemen van de patiënt met het vinden van de weg in leefge­bieden of omgeving (bijvoorbeeld verdwalen). Test formeel door tekenen en natekenen van een ontwerp, het rangschikken van puzzelstukjes, of door het tekenen van een kaart en het benoemen van belangrijke steden enzovoort. Houd rekening met ieder visueel gebrek dat het uitvoeren kan beïnvloeden.

0 Geen vermindering

1 Lichte vermindering zodat het globale ontwerp, de meeste details correct zijn; en/of weinig moeite om de weg te vinden in zijn/haar omgeving

2 Matig ernstige vermindering met verstoorde inschatting van het globale ontwerp en/of diverse fouten van details of onderdelen; en/of moet herhaald op de juiste richting gewezen worden om verdwalen te voorkomen in een nieuwere omgeving ondanks moeite om bekende objecten te lokaliseren in de directe omgeving

3 Zeer ernstige vermindering bij formeel testen; en/of herhaald zwerven of verdwalen in de omgeving

**DRS-R-98 facultatieve diagnostische items**

De volgende drie items kunnen worden gebruikt als steun bij de differentiatie van het delirium van andere stoornissen voor diagnostische en wetenschappelijke doeleinden.

**Chronologisch begin van de symptomen**

Scoor de abruptheid van het begin van de eerste symptomen van de stoornis of episode die op dit moment onderzocht wordt, niet de totale duur van de symptomen. Let hierbij uitsluitend op het begin van symptomen die toegeschreven worden aan een delirium, wanneer het delirium tegelijkertijd optreedt met een andere preëxistente psychiatrische stoornis. Bijvoorbeeld, wanneer de score wordt bepaald tijdens een delirante episode als gevolg van een overdosis bij een patiënt met een depressieve episode, scoor dan het begin van de delirante symptomen.

0 Geen significante verandering van het normale of lang bestaand standaard gedrag

1 Geleidelijk begin van symptomen, in het verloop van een periode van enkele weken tot een maand

2 Acute verandering van gedrag of persoonlijkheid, in het verloop van dagen tot een week

3 Abrupte verandering van gedrag, in het verloop van enkele uren tot een dag

**Wisselende ernst van symptomen**

Scoor het verergeren en verminderen van een enkel symptoom of een symptomencomplex gedurende het tijdsbestek waarop het onderzoek gericht is, Betreft meestal cognitie, affect, intensiteit van hallucinaties, denkstoornis of taalstoornis, Bedenk dat waarnemingsstoornissen meestal intermitterend optreden, maar kunnen samenkomen in een periode van grotere intensiteit, wanneer andere symptomen in ernst fluctueren,

0 Geen wisseling van ernstsymptomen

1 Intensiteit van symptomen wisselt wat betreft ernst in uren

2 Intensiteit van symptomen wisselt wat betreft ernst in minuten

**Lichamelijke aandoening**

Scoor de mate waarin een fysiologisch, somatisch of farmacologisch probleem specifiek aangewezen kan worden als oorzaak voor de symptomen, die onderzocht worden, Veel patiënten hebben zulke problemen, maar ze kunnen al of niet een causale relatie hebben met de symptomen die gescoord worden,

0 Niet aanwezig of actief

1 Aanwezigheid van een lichamelijke aandoening die de psychische toestand kan beïnvloeden

2 Geneesmiddel, infectie, metabole stoornis, CZS-laesie of ander somatisch probleem dat specifiek aangewezen kan worden als oorzaak voor het veranderd gedrag of psychische toestand,

SF-36

| **Hoe beoordeelt u uw gezondheid? (RAND-SF-36)**  In deze vragenlijst wordt naar uw gezondheid gevraagd. Wilt u elke vraag beantwoorden door het juiste hokje aan te kruisen. Wanneer u twijfelt over het antwoord op een vraag, probeer dan het antwoord te geven dat het meest van toepassing is. | | | | | | | | | | | | |
| --- | --- | --- | --- | --- | --- | --- | --- | --- | --- | --- | --- | --- |
| 1 | Wat vindt u, over het algemeen genomen, van uw gezondheid? |  |          |  | uitstekend  zeer goed  goed  matig  slecht | | | | | | | |
| 2 | *In vergelijking met een jaar geleden*, hoe zou u *nu* uw gezondheid in het algemeen beoordelen? |  |          |  | veel beter dan een jaar geleden  iets beter dan een jaar geleden  ongeveer hetzelfde als een jaar geleden  iets slechter dan een jaar geleden  veel slechter dan een jaar geleden | | | | | | | |
| 3 | De volgende vragen gaan over dagelijkse bezigheden. Wordt u door **uw gezondheid** op dit moment beperkt bij deze bezigheden? Zo ja, in welke mate? | | | | | | | | | | |  |
|  |  |  | **ja,**  **ernstig**  **beperkt** | | | |  | | **ja,**  **een beetje**  **beperkt** |  | **nee,**  **helemaal niet**  **beperkt** |  |
| a | *forse inspanning* zoals hardlopen, zware voorwerpen tillen, inspannend sporten |  |  | | | |  | |  |  |  |  |
| b | *matige inspanning* zoals het verplaatsen van een tafel, stofzuigen, fietsen. |  |  | | | |  | |  |  |  |  |
| c | tillen of boodschappen dragen |  |  | | | |  | |  |  |  |  |
| d | *een paar* trappen oplopen |  |  | | | |  | |  |  |  |  |
| e | *één* trap oplopen |  |  | | | |  | |  |  |  |  |
| f | buigen, knielen of bukken |  |  | | | |  | |  |  |  |  |
| g | *meer dan een kilometer* lopen |  |  | | | |  | |  |  |  |  |
| h | *een halve kilometer* lopen |  |  | | | |  | |  |  |  |  |
| i | *honderd meter* lopen |  |  | | | |  | |  |  |  |  |
| j | uzelf wassen of aankleden |  |  | | | |  | |  |  |  |  |
|  |  |  |  | | | |  | |  |  |  |  |
| 4 | Had u, ten gevolge van uw **lichamelijke gezondheid**, *de afgelopen 4 weken* één van de volgende problemen bij uw werk of andere dagelijkse bezigheden? | | | | | | | | | | |  |
|  |  | | | | |  | | **ja** | |  | **nee** |  |
| a | U heeft *minder tijd* kunnen besteden aan werk of andere bezigheden | | | | |  | |  | |  |  |  |
| b | U heeft *minder bereikt* dan u zou willen | | | | |  | |  | |  |  |  |
| c | U was beperkt in het *soort* werk of het soort bezigheden | | | | |  | |  | |  |  |  |
| d | U had moeite met het werk of andere bezigheden (het kostte u bijvoorbeeld extra inspanning) | | | | |  | |  | |  |  |  |

| 5 | Had u, ten gevolge van een **emotioneel probleem** (bijvoorbeeld doordat u zich depressief of angstig voelde), *de afgelopen 4 weken* één van de volgende problemen bij uw werk of andere dagelijkse bezigheden? | | | | |
| --- | --- | --- | --- | --- | --- |
|  |  |  | **ja** |  | **nee** |
| a | U heeft *minder tijd* kunnen besteden aan werk of andere bezigheden |  |  |  |  |
| b | U heeft *minder bereikt* dan u zou willen |  |  |  |  |
| c | U heeft het werk of andere bezigheden niet zo zorgvuldig gedaan als u gewend bent |  |  |  |  |

| 6 | In hoeverre heeft uw **lichamelijke gezondheid** of hebben uw **emotionele problemen** u *de afgelopen 4 weken* belemmerd in uw normale sociale bezigheden met gezin, vrienden, buren of anderen? | | | | | | |  |          | |  | | helemaal niet  enigszins  nogal  veel  heel erg veel | | | | |
| --- | --- | --- | --- | --- | --- | --- | --- | --- | --- | --- | --- | --- | --- | --- | --- | --- | --- |
| 7 | Hoeveel **pijn** had u *de afgelopen 4 weken*? | | | | | | |  |            | |  | | geen  heel licht  licht  nogal  ernstig  heel ernstig | | | | |
| 8 | In welke mate heeft **pijn** u *de afgelopen vier weken* belemmerd bij uw normale werkzaam­heden (zowel werk buitenshuis als huishoudelijk werk)? | | | | | | |  |          | |  | | helemaal niet  een klein beetje  nogal  veel  heel erg veel | | | | |
|  |  | | | | | | |  |  | |  | |  | | | | |
| 9 | Deze vragen gaan over hoe u zich *de afgelopen 4 weken* heeft **gevoeld**. Wilt u bij elke vraag het antwoord omcirkelen dat het beste aansluit bij hoe u zich heeft gevoeld. | | | | | | | | | | | | | | | | |
|  | Hoe vaak gedurende *de afgelopen 4 weken*: |  | **voort-**  **durend** |  | **meestal** |  | **vaak** | | |  | | **soms** | |  | **zelden** |  | **nooit** |
| a | voelde u zich levenslustig? |  |  |  |  |  |  | | |  | |  | |  |  |  |  |
| b | voelde u zich erg zenuwachtig? |  |  |  |  |  |  | | |  | |  | |  |  |  |  |
| c | zat u zo erg in de put dat niets u kon opvrolijken? |  |  |  |  |  |  | | |  | |  | |  |  |  |  |
| d | voelde u zich kalm en rustig? |  |  |  |  |  |  | | |  | |  | |  |  |  |  |
|  | Hoe vaak gedurende *de afgelopen 4 weken*: |  | **voort-**  **durend** |  | **meestal** |  | **vaak** | | |  | | **soms** | |  | **zelden** |  | **nooit** |
| e | voelde u zich erg energiek? |  |  |  |  |  |  | | |  | |  | |  |  |  |  |
| f | voelde u zich neerslachtig en somber? |  |  |  |  |  |  | | |  | |  | |  |  |  |  |
| g | voelde u zich uitgeblust? |  |  |  |  |  |  | | |  | |  | |  |  |  |  |
| h | voelde u zich gelukkig? |  |  |  |  |  |  | | |  | |  | |  |  |  |  |
| i | voelde u zich moe? |  |  |  |  |  |  | | |  | |  | |  |  |  |  |

| 10 | *Hoe vaak* hebben uw **lichamelijke gezondheid** of **emotionele problemen** gedurende *de afgelopen 4 weken* uw sociale activiteiten (zoals bezoek aan vrienden of naaste familiele­den) belemmerd? | | |  |          | | |  | voortdurend  meestal  soms  zelden  nooit | | | | | | |
| --- | --- | --- | --- | --- | --- | --- | --- | --- | --- | --- | --- | --- | --- | --- | --- |
| 11 | Wilt u het antwoord kiezen dat het beste weergeeft hoe juist of onjuist u elk van de volgende uitspraken voor uzelf vindt. | | | | | | | | | | | | | | |
|  |  |  | **volkomen juist** | | |  | **groten-**  **deels**  **juist** | | |  | **weet**  **ik niet** |  | **groten-**  **deels**  **onjuist** |  | **volkomen**  **onjuist** |
| a | Ik lijk gemakkelijker ziek te worden dan andere mensen |  |  | | |  |  | | |  |  |  |  |  |  |
| b | Ik ben net zo gezond als andere mensen die ik ken |  |  | | |  |  | | |  |  |  |  |  |  |
| c | Ik verwacht dat mijn gezondheid achteruit zal gaan |  |  | | |  |  | | |  |  |  |  |  |  |
| d | Mijn gezondheid is uitstekend |  |  | | |  |  | | |  |  |  |  |  |  |
